# Influenza vaccine hesitancy versus uptake in seven Chinese megacities: a cross-sectional survey

**DOI:** 10.1186/s40249-026-01445-6

**Published:** 2026-05-03

**Authors:** Tao Li, Qian Xiong, Peixin Wu

**Affiliations:** 1 School of Health Policy and Management, Chinese Academy of Medical Sciences and Peking Union Medical College, Beijing, 100730 China; 2 Peking Union Medical College Hospital, Beijing, China

**Keywords:** Influenza, Vaccine hesitancy, Vaccination coverage, Megacity, China

## Abstract

**Background:**

Influenza vaccine hesitancy remains a major barrier to achieving adequate vaccination coverage, particularly in densely populated urban areas with aging populations. Seven Chinese megacities are especially vulnerable to influenza transmission, yet evidence on drivers of vaccine hesitancy in these contexts remains limited. This study aimed to assess the prevalence and determinants of influenza vaccine hesitancy (IVH) among urban populations to inform strategies for improving vaccine uptake in China.

**Methods:**

We conducted a multi-city cross-sectional survey from March to June 2025 across seven Chinese megacities (Beijing, Shanghai, Guangzhou, Shenzhen, Tianjin, Chongqing, and Chengdu). Participants were selected using a stratified sampling strategy within communities; eligible participants were residents (aged ≥ 18 years) who had lived in the selected communities for at least six months. Data on demographic, socioeconomic, attitudinal factors, and behavioural factors were collected. Multivariable logistic regression was used to identify factors associated with IVH and pre-season influenza vaccine uptake.

**Results:**

A total of 8689 participants were included. Overall, 45% of participants were classified as vaccine hesitant, while the self-reported influenza vaccination coverage for the 2024 season was 30%. Substantial variation in hesitancy was observed across megacities. Older adults (≥ 65 years) were less likely to report IVH than younger adults (18–44 years) [adjusted odds ratio (*aOR*) = 0.72, 95% confidence interval (*CI*): 0.55–0.93] and more likely to be vaccinated (*aOR* = 1.63, 95% *CI*: 1.35–1.96). Psychosocial factors were the strongest predictors of hesitancy, particularly low perceived vaccine benefit (*aOR* = 11.18, 95% *CI*: 8.45–14.64) and low trust in health authorities (*aOR* = 17.13, 95% *CI*: 13.51–21.71). Vaccination uptake was primarily associated with behavioural factors, especially prior COVID-19 vaccination (*aOR* = 0.32, 95% *CI*: 0.25–0.39).

**Conclusions:**

Influenza vaccination in Chinese megacities is driven by a mix of demographic, psychosocial, and behavioural factors. Targeted strategies addressing both hesitancy and uptake are needed to improve coverage and reduce influenza transmission.

**Supplementary Information:**

The online version contains supplementary material available at 10.1186/s40249-026-01445-6.

## Background

Influenza vaccination is one of the most effective ways to prevent influenza infection and reduce associated severe illness and mortality [1]. Influenza could cause hundreds of thousands of infections annually and contribute substantially to the global disease burden [2, 3], which was responsible for 3 to 5 million cases of severe illness and 290,000 to 650,000 deaths annually [4, 5]. The administration of influenza vaccine stands out as a highly effective approach for safeguarding vaccinated individuals. It plays a pivotal role in fostering herd immunity within the population, which offers indirectly protection to unvaccinated individuals and reduces the overall public health burden of influenza epidemics [6–8]. Despite its importance, influenza vaccination coverage in China remains low, estimated at around 2.5% [9], well below World Health Organization (WHO) targets and the higher uptake observed in high-income countries such as the United States [10, 11]. This gap persists despite national recommendations that prioritize key groups, including children, older adults, and healthcare workers (HCWs) [12].

Vaccine hesitancy (VH) is defined as the delay in acceptance or refusal of vaccination despite the availability of vaccination services [13], and is a major factor limiting influenza vaccine uptake among adults in China [14]. VH is complex and context-specific, shaped by individual beliefs, social and group norms, and vaccine- or program-related factors [13]. Among adults in urban megacities, influenza vaccine hesitancy (IVH) remains substantial, driven by low confidence in vaccine safety and efficacy, complacency regarding disease risk, and barriers to convenient access [15]. However, in the post-COVID-19 era, public attitudes toward vaccination, including influenza vaccination, have shifted markedly, with greater awareness of vaccine importance and increased willingness to vaccinate; for instance, influenza vaccination coverage in the same areas of Shanghai increased from 11.8% to 17.7% following the pandemic [16].

China has undergone unprecedented urbanization, with over 600 million people migrating from rural to urban areas, leading to the emergence of numerous megacities with populations exceeding 10 million [17]. These megacities, including Beijing, Shanghai, Guangzhou, Shenzhen, Chengdu, Chongqing, and Tianjin, exhibit distinct characteristics shaped by their geographic location, economic specialization, and governance models. While residents in these megacities generally benefit from multi-channel healthcare education, improved healthcare access, and well-developed medical infrastructure, megacities also face challenges such as overcrowding and air pollution. Intra-urban inequalities, including disparities in green space, housing quality, and environmental exposure, might further increase the risk of respiratory infections such as influenza and shape vaccination behaviours [18]. Megacities, characterized by high population density, extensive mobility, and aging populations, can amplify transmission potential and increase the influenza disease burden compared with rural areas [19, 20]. In this context, influenza vaccination is one of the most effective and cost-efficient interventions to mitigate the health and economic impacts of influenza in megacities, providing both individual protection and urban public health resilience.

Understanding the prevalence and determinants of IVH in urban populations, particularly in megacities, is essential for designing effective vaccination strategies to improve influenza vaccine coverage in China. In this study, we conducted a cross-sectional survey across seven major Chinese megacities, which were selected based on population size reported in the 2020 Seventh National Census, in the post COVID-19 period. We aimed to assess post-pandemic changes in influenza vaccination behaviour, identify determinants of IVH, and provide evidence to guide targeted interventions to enhance influenza vaccine coverage in these urban settings. The findings are intended to inform evidence-based public health policies and tailored vaccination campaigns in urban China.

## Methods

### Study design and data collection

From March to June 2025, a cross-sectional survey was conducted in seven megacities in China, including Beijing, Shanghai, Chongqing, Tianjin, Guangzhou, Shenzhen, and Chengdu, to assess residents’ knowledge, attitudes, and behaviours related to IVH. A stratified random sampling strategy was applied across central districts in each megacity, with districts selected proportional to population size. Within selected districts, communities were chosen as survey sites using simple random sampling. Sample sizes were further allocated according to the local age and sex distribution to enhance demographic representativeness. Eligible participants were residents aged ≥ 18 years who had lived in the selected communities for at least six months. Within each selected community, eligible individuals were invited to participate through on-site recruitment until the target demographic quota was reached. Individuals who declined participation were not enrolled, and no additional information on non-respondents was available. No health-related eligibility restrictions were applied, as the survey was targeted at the general population. A flow chart illustrating the detailed sampling process was provided in Supplementary Figure S1. All participants provided informed consent prior to participation and received a small token of appreciation for their time.

Data were collected via offline questionnaires on the Wenjuanxing platform, administered face-to-face with participants, and local coordinators provided assistance to ensure completeness and accuracy. The questionnaire was pilot-tested among 116 participants to assess item clarity and face validity, and refined based on pilot feedback and expert panel review before use in the main survey. The sample size for each city was calculated based on standard formulas for cross-sectional surveys, accounting for expected variability, design effect, and potential non-response. The sample size for each megacity was calculated using the formula $n=\frac{{Z}^{2}\times p\left(1-p\right)}{{e}^{2}},$ assuming a 95% confidence level (Z = 1.96), an expected influenza vaccine hesitancy rate of 50% ($p=0.5$), and a margin of error ($e=5\mathrm{\%}$), yielding an initial sample of 384. After accounting for design effect (DEFF = 2.5) and 20% potential sample loss, the adjusted target sample size was 1152 per megacity, resulting in a total of 8064 participants. Stratified random sampling was applied across districts proportional to population size to ensure representativeness by age and sex based on population structure from the Seventh National Census (2020) [17]. A standardized quality control protocol was implemented, with city-specific staff training and real-time monitoring of data completeness and response quality to ensure consistency and integrity.

### Outcome measures

The primary outcome of this study was IVH, assessed using the statement: “I believe it is important to get the influenza vaccine annually*”*. Responses were recorded on a 5-point Likert scale ranging from “strongly disagree” to “strongly agree”. Participants responding “strongly disagree”, “disagree”, or “neutral” were classified as vaccine hesitancy, whereas those responding “agree” or “strongly agree” were classified as no/low vaccine hesitancy**.** This dichotomized variable was used for the main analyses. Although IVH is a multidimensional construct, this single-item measure served as a practical proxy for vaccination intention, consistent with prior population-based studies [21]. To assess the robustness of this classification, a sensitivity analysis was conducted in which “neutral” were instead classified as no/low vaccine hesitancy. The secondary outcome was self-reported influenza vaccine uptake during the 2024 season, reflecting actual vaccination behaviour in the previous season.

### Potential factors associated with IVH and pre-season influenza vaccine uptake

Potential factors associated with IVH and pre-season influenza vaccine uptake were examined across multiple domains, including sociodemographic characteristics, health-related factors, perceived influenza infection risk, confidence in health authorities, trust in the government’s preventive capacity, and vaccine-related beliefs (see Supplementary Table S2). Sociodemographic variables included age, gender, occupation, annual personal income, residential area, length of residence in the current city, and living status (classified as living alone or living with others). Health-related factors included chronic medical conditions, prior COVID-19 vaccination, and self-rated health status. Perceived influenza infection risk was assessed based on perceived susceptibility, perceived severity, and worry about contracting influenza during the upcoming season. Confidence in health authorities and trust in the government’s preventive capacity reflected respondents’ perceptions of the effectiveness of public health institutions in preventing infectious diseases. Vaccine-related beliefs included perceived vaccine safety, effectiveness, importance, and compatibility with personal values and social norms. Detailed item wording and coding are provided in Supplementary Table S2.

### Statistical analysis

Data analysis was conducted in three steps. First, logistic regression models were used to estimate city-specific probabilities of IVH and self-reported pre-season (2024) influenza vaccine uptake rate. Predicted proportions of IVH and vaccine coverage, along with 95% confidence intervals (*CI*s), were derived from the fitted logistic regression models. Second, multivariable logistic regression models were used to examine factors associated with IVH. Third, similar multivariable logistic regression models were applied to assess factors associated with pre-season influenza vaccine uptake in each megacity. All statistical tests were two-sided, with *P* < 0.05 considered statistically significant. Results were reported as adjusted odds ratios (*aOR*s) with 95% *CI*s.

All statistical analyses were conducted using R software (version 4.3.3; R Foundation for Statistical Computing, Vienna, Austria).

## Results

### Characteristics of recruited residents from seven megacities

In this survey, 9198 residents aged $\ge$ 18 years across seven megacities were approached in person. After quality control, 8689 (94%) questionnaires were deemed valid, with the number of valid questionnaires from each city as follows: Beijing (1285), Shanghai (1131), Guangzhou (1236), Shenzhen (1184), Tianjin (1265), Chongqing (1222), and Chengdu (1366). The age of the participants ranged from 18 to 94 years, with a mean age of 44 ± 16 years, and females accounted for 52% of the sample. Most participants (93.2%) reported living with family or others, while 6.8% lived alone. Around half of the residents (49.6%) resided in city centers, 34.3% in suburban areas, and 16.1% in outer suburban or other areas. About one-fifth (18.8%) of participants reported having chronic medical conditions. Most residents rated their health as good (59.6%) or fair (38.5%), with a mean self-rated health score of 7.75 ± 1.84. In terms of socioeconomic characteristics, 28.8% reported an annual household income of 10,000–49,999 CNY, while 17.5% earned less than 10,000 CNY. In addition, 90.5% of participants were fully vaccinated against COVID-19, and 65.8% of them expressed high trust in the government’s preventive capacity (Table 1).

**Table 1:** Characteristics of participants and distribution of influenza vaccine hesitancy and uptake among residents in seven megacities in China, March to June 2025

Characteristics	Overall (*n* = 8689) *n* (%)	Influenza vaccine hesitancy			Pre-season influenza vaccination status		
		No/low vaccine hesitancy (*n* = 4761) *n* (%)	Vaccine hesitancy (*n* = 3928) *n* (%)	*P*-value	Not vaccinated *(n* = 6116) *n* (%)	Vaccinated (*n* = 2573) *n* (%)	*P*-value
Sex				< 0.01			0.05
Male	4128 (48.0%)	2130 (44.7%)	1998 (50.9%)		2948 (48.2%)	1180 (45.9%)	
Female	4561 (52.0%)	2631 (55.3%)	1930 (49.1%)		3168 (51.8%)	1393 (54.1%)	
Age group				< 0.01			0.02
Young adults (18–44 years)	4791 (55.1%)	2693 (56.6%)	2098 (53.4%)		3371 (55.1%)	1420 (55.2%)	
Adults (45–64 years)	2662 (30.6%)	1468 (30.8%)	1194 (30.4%)		1912 (31.3%)	750 (29.1%)	
Older adults (≥ 65 years)	1236 (14.2%)	600 (12.6%)	636 (16.2%)		833 (13.6%)	403 (15.7%)	
Type of residence				0.01			< 0.01
City center	4308 (49.6%)	2420 (50.8%)	1888 (48.1%)		3008 (49.2%)	1300 (50.5%)	
Suburban	2977 (34.3%)	1613 (33.9%)	1364 (34.7%)		2047 (33.5%)	930 (36.1%)	
Outer Suburban and other	1404 (16.2%)	728 (15.3%)	676 (17.2%)		1061 (17.3%)	343 (13.3%)	
Education				< 0.01			0.07
Primary school or below	108 (1.2%)	43 (0.9%)	65 (1.7%)		81 (1.3%)	27 (1.0%)	
Middle school/Junior high	1429 (16.4%)	662 (13.9%)	767 (19.5%)		1033 (16.9%)	396 (15.4%)	
High school/Vocational	3287 (37.8%)	1783 (37.4%)	1504 (38.3%)		2338 (38.2%)	949 (36.9%)	
Bachelor's degree	3363 (38.7%)	1973 (41.4%)	1390 (35.4%)		2313 (37.8%)	1050 (40.8%)	
Master's degree or above	502 (5.8%)	300 (6.3%)	202 (5.1%)		351 (5.7%)	151 (5.9%)	
Occupation				< 0.01			< 0.01
Professional	1910 (22.0%)	1377 (28.9%)	533 (13.6%)		1178 (19.3%)	732 (28.4%)	
Clerical and service worker	3226 (37.1%)	1655 (34.8%)	1571 (40.0%)		2385 (39.0%)	841 (32.7%)	
Production worker	1027 (11.8%)	473 (9.9%)	554 (14.1%)		729 (11.9%)	298 (11.6%)	
Student	317 (3.6%)	170 (3.6%)	147 (3.7%)		214 (3.5%)	103 (4.0%)	
Homemaker/Retired/Not employed nor schooling	1815 (20.9%)	897 (18.8%)	918 (23.4%)		1314 (21.5%)	501 (19.5%)	
Other	394 (4.5%)	189 (4.0%)	205 (5.2%)		296 (4.8%)	98 (3.8%)	
Annual income				< 0.01			0.30
< 10,000 CNY	1524 (17.5%)	790 (16.6%)	734 (18.7%)		1054 (17.2%)	470 (18.3%)	
10,000–49,999 CNY	2501 (28.8%)	1318 (27.7%)	1183 (30.1%)		1772 (29.0%)	729 (28.3%)	
50,000–99,999 CNY	2080 (23.9%)	1192 (25.0%)	888 (22.6%)		1,481 (24.2%)	599 (23.3%)	
100,000–199,999 CNY	1444 (16.6%)	869 (18.2%)	575 (14.6%)		1025 (16.8%)	419 (16.3%)	
200,000–499,999 CNY	484 (5.6%)	283 (5.9%)	201 (5.1%)		345 (5.6%)	139 (5.4%)	
≥ 500,000 CNY	162 (1.9%)	96 (2.0%)	66 (1.7%)		103 (1.7%)	59 (2.3%)	
Unclear/Unknown	494 (5.7%)	213 (4.5%)	281 (7.2%)		336 (5.5%)	158 (6.1%)	
Chronic medical condition				0.02			0.80
Yes	1633 (18.8%)	838 (17.6%)	795 (20.2%)		1145 (18.7%)	488 (19.0%)	
No	7056 (81.2%)	3923 (82.4%)	3133 (79.8%)		4971 (81.3%)	2085 (81.0%)	
Fully vaccinated against COVID-19				< 0.01			< 0.01
Yes	7862 (90.5%)	4413 (92.7%)	3449 (87.8%)		5391 (88.1%)	2,471 (96.0%)	
No	827 (9.5%)	348 (7.3%)	479 (12.2%)		725 (11.9%)	102 (4.0%)	
Length of residence				0.07			0.30
0.5–1 years	6849 (78.8%)	3805 (79.9%)	3044 (77.5%)		4784 (78.2%)	2065 (80.3%)	
1–2 years	284 (3.3%)	139 (2.9%)	145 (3.7%)		203 (3.3%)	81 (3.1%)	
2–3 years	455 (5.2%)	236 (5.0%)	219 (5.6%)		329 (5.4%)	126 (4.9%)	
3–5 years	577 (6.6%)	286 (6.0%)	291 (7.4%)		416 (6.8%)	161 (6.3%)	
≥ 5 years	524 (6.0%)	295 (6.2%)	229 (5.8%)		384 (6.3%)	140 (5.4%)	
Living status				0.60			< 0.01
Alone	591 (6.8%)	317 (6.7%)	274 (7.0%)		455 (7.4%)	136 (5.3%)	
With others	8098 (93.2%)	4444 (93.3%)	3654 (93.0%)		5661 (92.6%)	2437 (94.7%)	
Self-rated health				< 0.01			0.08
Poor	168 (1.9%)	68 (1.4%)	100 (2.5%)		123 (2.0%)	45 (1.7%)	
Fair	3344 (38.5%)	1624 (34.1%)	1720 (43.8%)		2395 (39.2%)	949 (36.9%)	
Good	5177 (59.6%)	3069 (64.5%)	2108 (53.7%)		3598 (58.8%)	1579 (61.4%)	
Trust in the government’s preventive capacity				< 0.01			< 0.01
High	5719 (65.8%)	4,227 (88.8%)	1492 (38.0%)		3859 (63.1%)	1860 (72.3%)	
Low	2970 (34.2%)	534 (11.2%)	2436 (62.0%)		2257 (36.9%)	713 (27.7%)	

^*^indicated *P* < 0.05, statistically significant

### Proportion of influenza vaccine hesitancy and pre-season influenza vaccination coverage by city

**Fig. 1:**
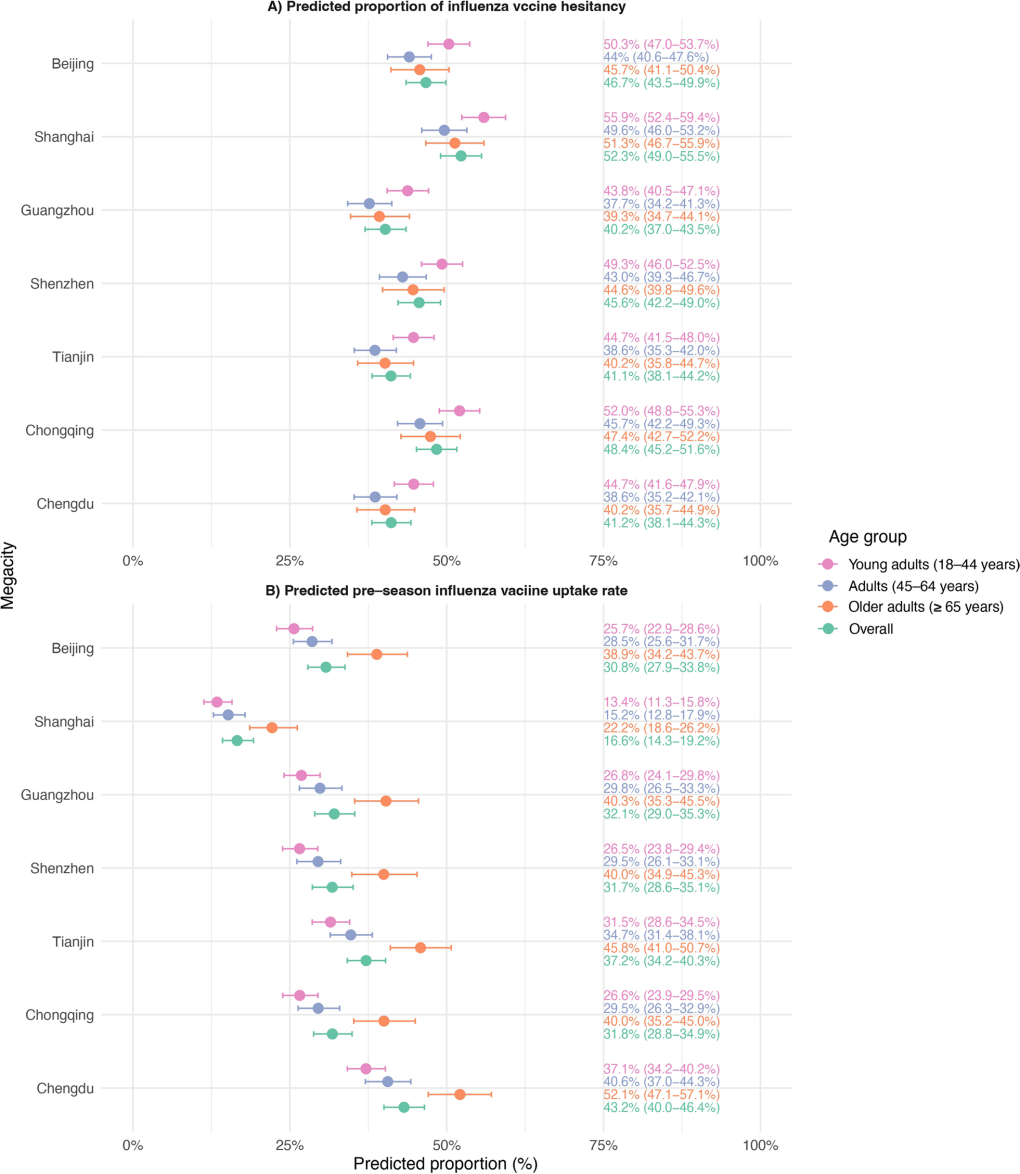
Predicted probabilities of influenza vaccine hesitancy and pre-season influenza vaccine uptake across seven megacities, stratified by age group. The forest plot shows survey-weighted predicted probabilities with 95% CI for each city, stratified by age group: young adults (18–44 years), adults (45–64 years), and older adults ($\ge$ 65 years). The left-aligned text column indicated the predicted probability (%) with its 95% CI. Points represented the predicted probabilities, and horizontal bars represented the 95% CIs. Age groups are color-coded, as indicated in the legend. Panels were separated by outcome: influenza vaccine hesitancy and pre-season influenza vaccine uptake (receipt of influenza vaccine in 2024)

Logistics regression models were used to estimate predicted proportions of IVH and pre-season vaccination coverage across seven Chinese megacities (Table S4). Overall, substantial inter-city variation was observed. The predicted proportion of vaccine hesitancy ranged from 40.2% (95% *CI*: 37.0–43.5%) in Guangzhou to 52.3% **(**95% *CI*: 49.0–55.5%) in Shanghai. Modest inter-city variation was observed, with relatively lower estimates in Tianjin (41.1%), Guangzhou (40.2%), and Chengdu (41.2%), and higher estimates in Shanghai (52.3%) and Chongqing (48.4%). Across all cities, younger adults (18–44 years) consistently exhibited higher levels of VH than older adults (≥ 65 years) (Fig. 1 and supplementary material Table S4). For pre-season influenza vaccination coverage also varied considerably by city, ranging from 16.6% (95% *CI*: 14.3–19.2%) in Shanghai to 43.2% (95% *CI*: 40.0–46.4%) in Chengdu. Older adults (≥ 65 years) consistently demonstrated higher influenza vaccine uptake than younger adults (18–44 years), with coverage reaching 52.1% (95% *CI*: 47.1–57.1%) in Chengdu and 45.8% (95% *CI*: 41.0–50.7%) in Tianjin. In contrast, coverage among young adults was below 30% in most megacities (Fig. 1 and supplementary material Table S4). Across seven megacities, the overall vaccination coverage was 29.6% (95% *CI*: 28.7–30.6%)**,** with city-level estimates ranging from 14.5% in Shanghai to 38.7% in Chengdu (supplementary material Table S5). We found that individuals who had received a pre-season influenza vaccine were significantly less likely to report IVH (*OR* = 0.35, 95% *CI*: 0.32–0.39).

### Determinants of influenza vaccine hesitancy and uptake across seven megacities in China

In binary logistic regression analysis across seven megacities, sociodemographic and behavioural factors were found to be associated with IVH and uptake. Older adults (≥ 65 years) were statistically significantly less likely to be hesitant (*aOR* = 0.72, 95% *CI*: 0.55–0.93) and more likely to uptake the influenza vaccine (*aOR* = 1.63, 95% *CI*: 1.35–1.96) compared to younger adults aged 18–44 years. While female was also statistically significantly less likely to be hesitant (*aOR* = 0.83, 95% *CI*: 0.72–0.94) compared to male, this attitudinal difference did not translate into higher vaccine uptake rates. Compared with professionals, individuals in non-professional occupations (including clerical, service, and production workers) and the unemployed had significantly higher odds of vaccine hesitancy (*aORs* ranged from 1.70 to 2.05) and lower odds of vaccine uptake (*aORs*: 0.56–0.72). Household composition played a facilitative role in behaviour enactment; living with others was statistically significantly associated with lower IVH and a 51% higher likelihood of vaccine uptake compared with living alone. Individuals who were not fully vaccinated against COVID-19 were statistically significantly more likely to report IVH (*aOR* = 1.49, 95% *CI*: 1.19–1.85) and less likely to have received influenza vaccination (*aOR* = 0.32, 95% *CI*: 0.25–0.39). Participants with low perceived vaccine benefit had higher odds of IVH (*aOR* = 11.18, 95% *CI*: 8.45–14.64), as did those with low trust in health authorities (*aOR* = 17.13, 95% *CI*: 13.51–21.71) and low trust in government’s preventive capacity (*aOR* = 1.41, 95% *CI*: 1.17–1.70) (Table 2). In sensitivity analyses, we used an alternative definition of hesitancy, in which “neutral” was re-classified as non-hesitant, showed that most associations remained consistent in direction and statistical significance, including occupation (clerical/service workers: *aOR* = 2.21; production workers: *aOR* = 2.37; students: *aOR* = 1.76; homemakers/retired/unemployed: *aOR* = 1.97; other: *aOR* = 2.78; all *P* < 0.05), COVID-19 vaccination status (unvaccinated: *aOR* = 1.42, *P* < 0.01), perceived infection risk (medium: *aOR* = 0.60; high: *aOR* = 1.24; both *P* < 0.01), perceived vaccine benefit (low: aOR = 2.08, *P* < 0.01), and trust in health authorities (low: *aOR* = 5.28, *P* < 0.01) (Supplementary Table S11).

**Table 2:** Multivariable logistic regression analysis of factors associated with influenza vaccine hesitancy and uptake among residents (aged ≥ 18 years) in seven megacities in Chinese mainland

Variables	Vaccine hesitancy		Vaccine uptake	
	a*OR* (95% *CI*)	*P*-value	a*OR* (95% *CI*)	*P-*value
Sex				
Male	Reference			
Female	0.83 (0.72, 0.94)	< 0.01	1.07 (0.97, 1.18)	0.16
Age group				
Young adults (18–44 years)	Reference			
Adults (45–64 years)	0.83 (0.71, 0.98)	0.03	1.00 (0.89, 1.13)	1.00
Older adults (≥ 65 years)	0.72 (0.55, 0.93)	0.01	1.63 (1.35, 1.96)	< 0.01
Residence type				
City center	Reference			
Suburban	1.14 (0.99, 1.32)	0.06	1.06 (0.95, 1.17)	0.30
Outer suburban and other	1.37 (1.14, 1.64)	< 0.01	0.74 (0.64, 0.85)	< 0.01
Occupation				
Professional	Reference			
Clerical and service worker	2.05 (1.72, 2.45)	< 0.01	0.63 (0.55, 0.71)	< 0.01
Production worker	1.70 (1.30, 2.22)	< 0.01	0.72 (0.60, 0.86)	< 0.01
Student	2.09 (1.43, 3.07)	< 0.01	0.92 (0.70, 1.22)	0.57
Homemaker/Retired/Not employed nor schooling	1.82 (1.43, 2.33)	< 0.01	0.56 (0.47, 0.67)	< 0.01
Other	1.83 (1.30, 2.59)	< 0.01	0.57 (0.44, 0.74)	< 0.01
Annual income				
< 10,000 CNY	Reference			
10,000–49,999 CNY	1.14 (0.92, 1.42)	0.22	0.91 (0.78, 1.06)	0.21
50,000–99,999 CNY	1.03 (0.82, 1.29)	0.79	0.89 (0.76, 1.04)	0.15
100,000–199,999 CNY	1.20 (0.94, 1.53)	0.14	0.84 (0.71, 1.00)	0.05
200,000–499,999 CNY	1.21 (0.89, 1.66)	0.23	0.82 (0.64, 1.05)	0.11
≥ 500,000 CNY	0.87 (0.52, 1.44)	0.59	1.21 (0.85, 1.73)	0.28
Unclear/Unknown	1.25 (0.91, 1.73)	0.17	1.13 (0.89, 1.42)	0.31
Chronic Medical condition				
Yes	Reference			
No	1.04 (0.85, 1.26)	0.72	0.94 (0.82, 1.08)	0.39
Self-rated health				
Poor	Reference			
Fair	0.91 (0.60, 1.39)	0.67	1.12 (0.87, 1.44)	0.39
Good	0.99 (0.76, 1.28)	0.94	0.94 (0.80, 1.10)	0.43
Living status				
Living alone	Reference			
Living with others	0.81 (0.64, 1.04)	0.1	1.51 (1.23, 1.86)	< 0.01
Fully COVID-19 vaccination*				
Yes	Reference			
No	1.49 (1.19, 1.85)	< 0.01	0.32 (0.25, 0.39)	< 0.01
Perceived infection risk of influenza				
Low	Reference			
Medium	0.68 (0.59, 0.79)	< 0.01	0.98 (0.88, 1.08)	0.64
High	0.78 (0.70, 0.88)	< 0.01	1.13 (1.04, 1.23)	< 0.01
Knowledge of influenza vaccine				
High	Reference			
Low	1.94 (1.61, 2.34)	< 0.01	0.62 (0.54, 0.70)	< 0.01
Vaccine confidence				
High	Reference			
Low	2.08 (1.77, 2.44)	< 0.01	0.94 (0.83, 1.07)	0.36
Perceived vaccine benefit				
High	Reference			
Low	11.18 (8.45, 14.64)	< 0.01	1.06 (0.89, 1.26)	0.5
Trust in health authorities				
High	Reference			
Low	17.13 (13.51, 21.71)	< 0.01	0.57 (0.49, 0.68)	< 0.01
Trust in government’s preventive capacity				
High	Reference			
Low	1.41 (1.17, 1.70)	< 0.01	1.13 (0.98, 1.31)	0.09
Preference for infection-derived immunity				
High	Reference			
Low	2.28 (1.96, 2.67)	< 0.01	0.78 (0.69, 0.88)	< 0.01

^*^Full COVID-19 vaccination was defined according to national guidelines as receipt of at least three doses of an inactivated COVID-19 vaccine during pandemic period

### Determinants of vaccine hesitancy and uptake by city

**Fig. 2:**
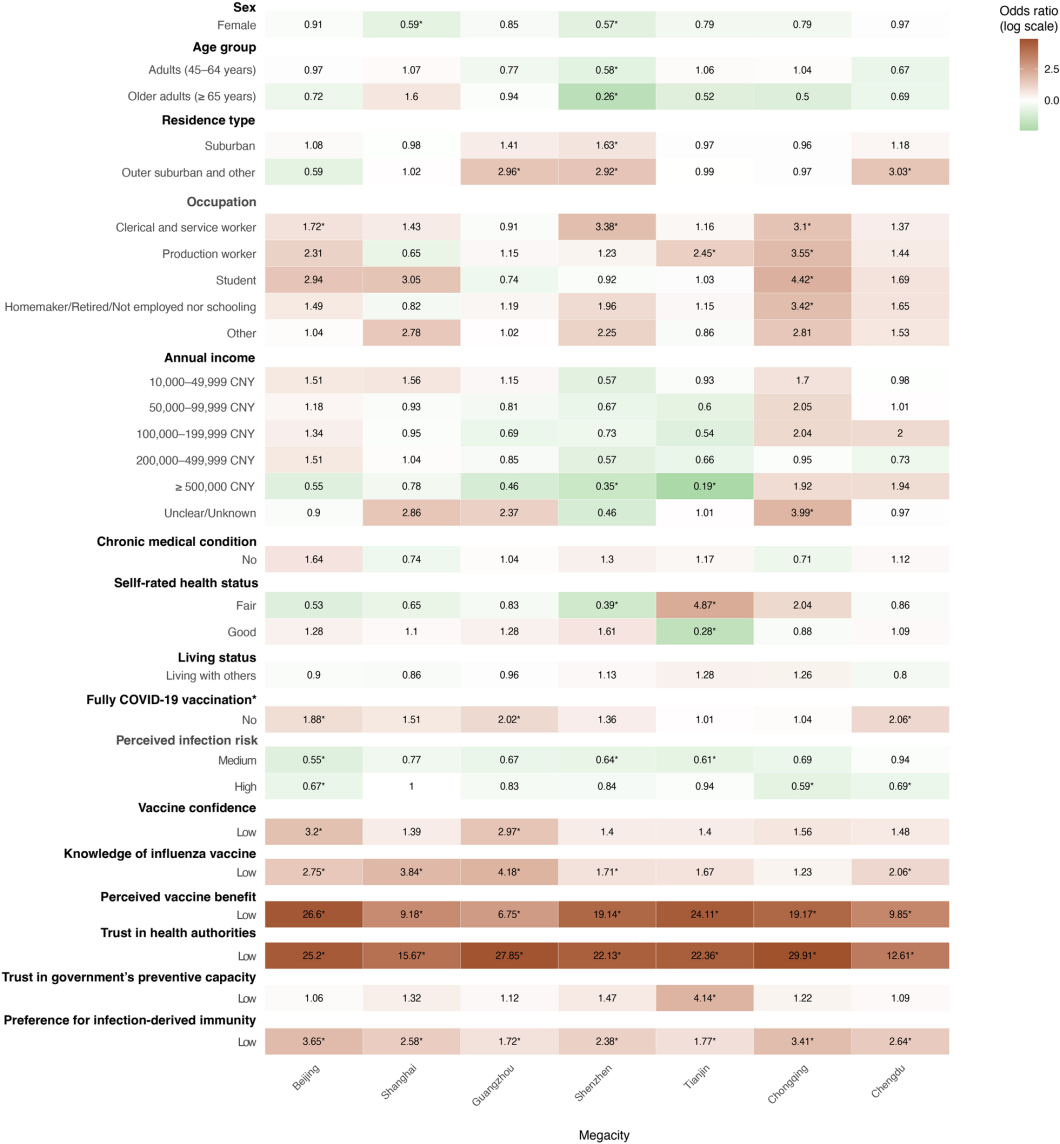
Multivariable logistic regression analysis of potential factors associated with influenza vaccine hesitancy among residents in seven megacities in China. Numbers in boxes shew adjusted odds ratios (a*ORs*), and numbers in black accompanied by an asterisk (“*”) indicated statistically significant. Green indicates factors associated with lower odds of vaccine hesitancy, whereas red indicated factors associated with higher odds of vaccine hesitancy

We then applied city-stratified binary logistic regression models, which revealed regional heterogeneity in the determinants of vaccination attitude and behaviour. Geographical and psychosocial factors were predominantly associated with IVH. In southern and western megacities, residential type was a critical determinant. Living in suburban or outer areas was statistically significantly associated with higher IVH in Guangzhou *(aOR* = 2.96, 95% *CI*: 1.70–5.14) and Shenzhen (*aOR* = 2.92, 95% *CI*: 1.40–6.09). Occupational disparities were particularly pronounced in Chongqing, where all non-professional groups reported significantly higher hesitancy compared with professionals, including clerical/service workers (*aOR* = 3.10, 95% *CI*: 1.78–5.38), production workers (*aOR* = 3.55, 95% *CI*: 1.75–7.22), and homemakers/retired/unemployed (*aOR* = 3.42, 95% *CI*: 1.71–6.83). Attitudinal barriers were most evident in northern municipalities, where low perceived vaccine benefit was associated with exceptionally high odds of IVH in Beijing (*aOR* = 26.6, 95% *CI*: 8.77–80.68) and Tianjin (*aOR* = 24.11, 95% *CI*: 9.85–59.01). Low trust in health authorities was consistently associated with higher odds of IVH across all seven megacities (*aOR*: 12.61–29.91; all *P* < 0.01). Beliefs in infection-derived immunity was also associated with VH across megacities, but the direction differed between primary and sensitivity analyses (e.g., Beijing: *aOR* = 3.65, 95% *CI*: 2.33–5.74; Chengdu: *aOR* = 2.64, 95% *CI*: 1.70–4.09), indicating that results are sensitive to the definition of IVH (Fig. 2, supplementary Table S7, Table S9, and Table S11).

**Fig. 3:**
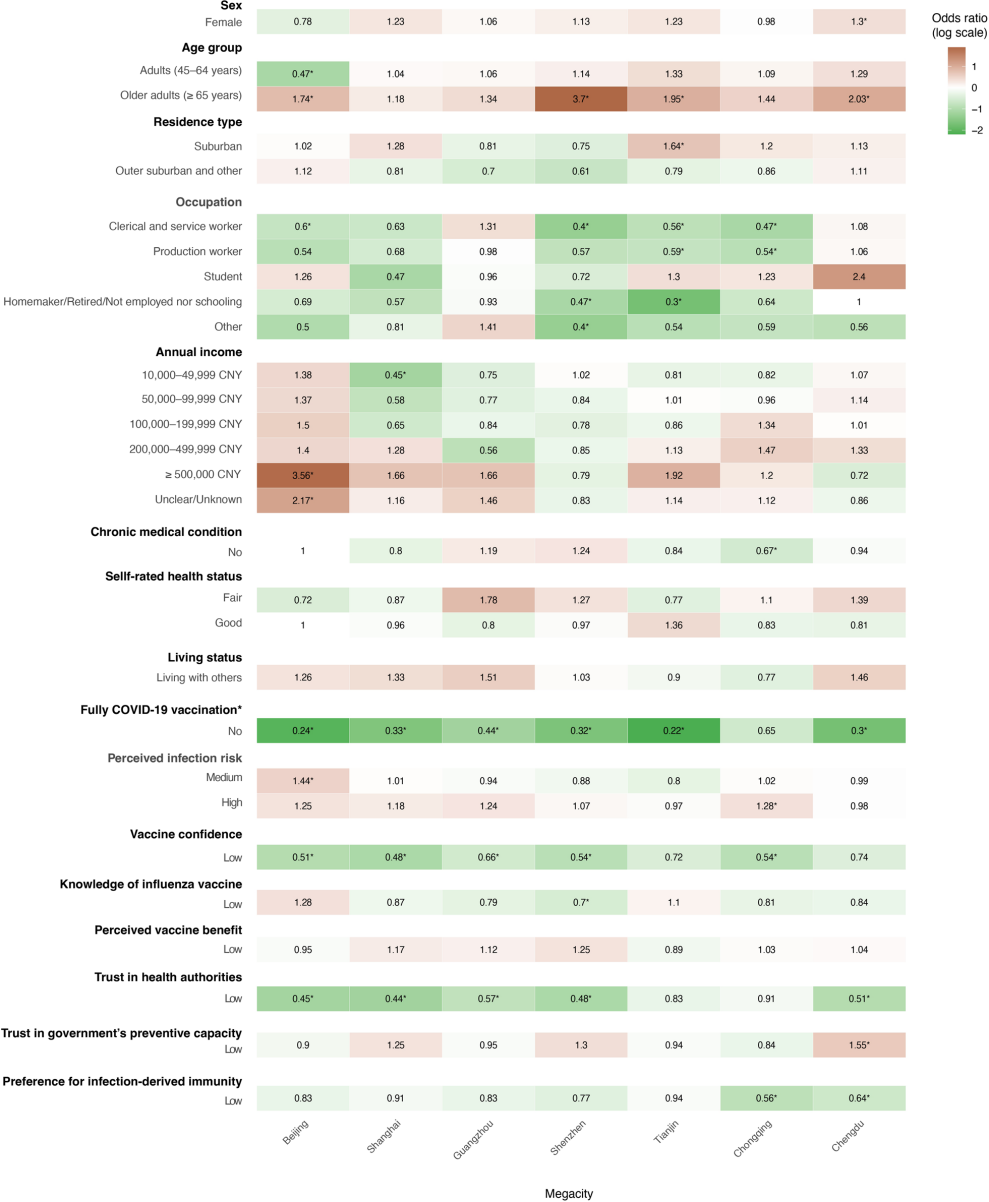
Multivariable logistic regression analysis of factors associated with influenza vaccine uptake among residents in seven megacities in China. Numbers within the boxes represent adjusted odds ratios (a*OR*s); black values with an asterisk (*) indicated statistical significance. Green indicates factors associated with higher odds of having been vaccinated, whereas red indicates factors associated with lower odds of having been vaccinated

However, the factors driving actual vaccine uptake differed from those associated with IVH, highlighting a gap between intention and behaviour. The association between COVID-19 and influenza vaccination remained robust at the city level. In six of the seven megacities (Beijing, Shanghai, Guangzhou, Shenzhen, Tianjin, and Chengdu), incomplete COVID-19 vaccination status was independently associated with a 56–78% reduction in the odds of influenza vaccine uptake. Income showed a unique gradient in Beijing, where high earners ($\ge$ 500,000 CNY) were over three times more likely to be vaccinated, while this pattern was not observed in other megacities. Occupational differences also remained significant in Beijing, Shenzhen, Tianjin, and Chongqing, where non-professional workers consistently had lower uptake rates. For example, clerical/service workers (Beijing: *aOR* = 0.60, 95% *CI*: 0.41–0.87; Shenzhen: *aOR* = 0.40, 95% *CI*: 0.28–0.58), production workers (Tianjin: *aOR* = 0.59, 95% *CI*: 0.38–0.90; Chongqing: *aOR* = 0.54, 95% *CI*: 0.34–0.86), and homemakers/retired/unemployed (Shenzhen: *aOR* = 0.47, 95% *CI*: 0.28–0.81; Tianjin: *aOR* = 0.30, 95% *CI*: 0.19–0.47), all showed lower odds of uptake. For psychosocial factors, unlike VH, perceived vaccine benefit was generally not a significant predictor of uptake across megacities (all *P* > 0.05 in stratified models). Instead, low vaccine knowledge was statistically significantly associated with reduced vaccine uptake in Beijing (*aOR* = 0.51, 95% *CI*: 0.33–0.78), Shanghai (*aOR* = 0.48, 95% *CI*: 0.29–0.77, p < 0.01), Guangzhou (*aOR* = 0.66, 95% *CI*: 0.48–0.90), and Shenzhen (*aOR* = 0.54, 95% *CI*: 0.39–0.76) (Fig. 3, supplementary Table S8, and Table S10).

## Discussion

Our multi-city survey provided a comprehensive overview of IVH and uptake among adults aged $\ge$ 18 years in seven Chinese megacities. Our findings revealed substantial inter-city heterogeneity, as well as important sociodemographic, behavioural, and perceptual determinants of vaccine attitudes and behaviours. The findings revealed a concerning gap between public health goals and reality: IVH remained high, with nearly half of respondents expressing reluctance, while pre-season vaccination coverage was suboptimal at approximately 30%. These estimates aligned with previous national surveys in China, which have historically reported low seasonal influenza vaccination coverage, reaching as low as 9.4% in general population [22].

The geographic and demographic disparities were observed across seven Chinese megacities. Residents in western and inland cities (e.g., Chengdu and Chongqing) demonstrated higher vaccine uptake and lower IVH, whereas those in more economically developed eastern cities (e.g., Shanghai and Beijing) demonstrated the opposite pattern. These differences might be driven, in part, by population mobility, which might disrupt continuity of healthcare messaging and weaken engagement with vaccination programs [23]. In terms of demographic disparities, we found that younger adults aged 18–44 years consistently exhibited higher IVH and lower uptake across all megacities, highlighting the need to better target this group in future vaccination campaigns. In contrast, older adults aged ≥ 65 years showed lower IVH and higher vaccine uptake, likely attributable to greater risk perception, high vaccine confidence [24], and more frequent interactions with healthcare services [25]. We also found that residents in suburban or outer areas of Shenzhen, Guangzhou, and Chengdu were more hesitant than those in city centres, in line with previous studies in China [15]. This urban-suburban gap might reflect differences in healthcare availability, convenience of vaccination sites, and exposure to public-health campaigns [15, 26]. Inter-city heterogeneity might be driven by variations in local health system organization, service delivery models, and municipal vaccination policies, including the provision of free influenza vaccination programs for priority populations in some megacities.

Beyond geographic and demographic factors, psychological and attitudinal variables were the most consistent predictors of both IVH and uptake across seven Chinese megacities. Participants who were fully vaccinated against COVID-19 were significantly more likely to receive the influenza vaccine and exhibited markedly lower levels of IVH, indicating a strong “cross-vaccine” behavioural consistency, suggesting that individuals who engaged with one public health intervention (COVID-19 shots) were more likely to engage with others. Our finding was consistent with several previous studies [27], however, some studies have also reported a negative relationship between COVID-19 or influenza vaccination behaviour and VH [28]. Similarly, individuals who received pre-season influenza vaccination were more likely to report lower IVH in the following year, suggesting a reinforcing cycle of positive vaccine attitudes and behaviours over time, consistent with a recent study conducted among older adults in China [29]. Furthermore, participants with lower perceived vaccine benefit or lower trust in health authorities had substantially higher odds of IVH, while those with greater vaccine confidence and trust in government’s preventive capacity were more likely to be vaccinated. This aligned with previous studies showing that lower institutional trust is associated with increased IVH [21, 30, 31]. However, although perceived vaccine benefits were associated with lower IVH, this did not consistently translate into higher vaccine uptake in stratified analyses. This discrepancy might reflect the influence of structural and access-related barriers, such as vaccination cost, service availability, and workplace policies, which were not captured in our survey but might limit the translation of positive attitudes into actual vaccination behaviour [32].

Overall, our findings underscored that a "one-size-fits-all" strategy is insufficient for urban China, and that multifaceted, city-specific strategies were needed to address IVH and improve influenza vaccine coverage. We observed suboptimal influenza vaccine uptake rate alongside persistently high levels of IVH, particularly among younger adults, residents in non-central urban districts, and those with lower trust in health authorities. Evidence-informed strategies, such as transparent risk communication, engagement of trusted community figures, and improved access to affordable or free vaccination, could play a critical role in enhancing vaccine acceptance. Community-based multifaceted programs have been shown to increase vaccine uptake by addressing knowledge gaps, building trust, and reducing financial and logistical barriers [33, 34]. Given that COVID-19 vaccination history was an important determinant of influenza uptake, co-administration strategies or "bundled" health messaging might be effective in improving the influenza vaccine coverage.

There were several limitations in this study. First, pre-season influenza vaccination status was self-reported and might be subject to recall bias. Second, the cross-sectional design precluded causal inference. Third, although the seven megacities included represent major Chinese megacities, the findings might not be generalizable to rural areas or smaller cities. Fourth, the reliance on self-reported data collected through a single survey instrument might introduce common-method variance, with responses potentially influenced by social desirability or consistency bias. Fifth, IVH was measured using a single attitudinal item reflecting perceived importance of annual influenza vaccination, which, although informative, might not fully reflect the multidimensional nature of IVH, including confidence, complacency, and convenience. Sixth, despite comprehensive assessment of attitudinal factors, unmeasured contextual factors, such as local policy implementation, healthcare infrastructure, and access barriers, might also influence IVH and uptake. Finally, we did not collect data on participants’ information sources, exposure to misinformation, or the underlying socio-political and cultural drivers of trust in health authorities. These unmeasured factors likely contribute to the observed heterogeneity in trust across different megacities and age groups, particularly among younger adults. Future research is needed to incorporate these dimensions to better understand the mechanisms influencing public trust and to address the gaps identified in our study.

## Conclusions

IVH remained prevalent across seven Chinese megacities, with demographic, socioeconomic, attitudinal, and behavioural factors shaping both hesitancy and vaccine uptake. Older adults and residents in western cities showed relatively higher vaccine acceptance, whereas younger adults and residents in eastern metropolitan areas were more reluctant. Strengthening public confidence in vaccines, building trust in health authorities, and addressing geographic and socioeconomic barriers are essential to improving influenza vaccination coverage and enhancing pandemic preparedness. Our findings provide a comprehensive assessment of pre-season influenza vaccination coverage and hesitancy, offering evidence to guide targeted public health interventions.

## Supplementary Information

Additional file 1.

## Data Availability

The survey data that support the findings of this study are available from the corresponding author upon reasonable request.
