# Correction: Influenza vaccine hesitancy versus uptake in seven Chinese megacities: a cross-sectional survey

**DOI:** 10.1186/s40249-026-01476-z

**Published:** 2026-07-15

**Authors:** Tao Li, Qian Xiong, Peixin Wu

**Affiliations:** 1 School of Health Policy and Management, Chinese Academy of Medical Sciences and Peking Union Medical College, Beijing, 100730 China; 2 Peking Union Medical College Hospital, Beijing, China

**Correction: Infectious Diseases of Poverty (2026) 15:48 **10.1186/s40249-026-01445-6.

Following the publication of the original article [1], it is reported that symbol “ ≥ ” is missing in the category “Annual income ≥ 500,000 CNY” in Table 1. The error was introduced during production, and has now been corrected.

The original article [1] has been updated.
