# Supplementary material for: Influenza vaccine hesitancy versus uptake in seven Chinese megacities: a cross-sectional survey
Source: Infect Dis Poverty. 2026 May 3;15:48. doi: 10.1186/s40249-026-01445-6 (PMC13137521; doi:10.1186/s40249-026-01445-6)
Supplement: Supplementary file 1 — Additional file 1. [file 40249_2026_1445_MOESM1_ESM.docx]

**Supplementary material**

**Influenza vaccine hesitancy versus uptake in seven Chinese megacities: a cross–sectional survey**

Table S1. Items used to assess influenza vaccine hesitancy.

| **Outcomes** | **Question / Item wording** | **Response options** | **Coding / Notes** |
| --- | --- | --- | --- |
| Primary outcome | How much you agree or disagree with the following statement?  I believe it is important to get the influenza vaccine annually. | 1 = Strongly disagree 2 = Disagree 3 = Neutral 4 = Agree 5 = Strongly agree | This item was used as a key indicator of hesitancy. Participants selecting 1–2 (“Strongly disagree” or “Disagree”) were classified as 'hesitant,' 3 (“Neutral”) was treated as hesitant in the main analysis and as non–hesitant in sensitivity analyses, and 4–5 (“Agree” or “Strongly agree”) were classified as 'non–hesitant. |
| Secondary outcome | “Did you receive the influenza vaccine in the most recent flu season (2024) prior to this survey?” | Yes / No | This item was used as a key indicator of actual vaccination behavior. |

Table S2. **List of potential factors associated with COVID–19 vaccine uptake and hesitancy**

| **Variable** | **Variable Category** | **Type** | **Categories / Coding** | **Reference** |
| --- | --- | --- | --- | --- |
| Age | Sociodemographic | Ordinal | Ordinal: Aged ≥18 years (Used in descriptive analysis).   Transition: Young adult (18–44 years), Adult (45–64 years), Older adult (≥ 65 years) (Used in both descriptive and regression analysis). | Adult (45–64 years) |
| Sex | Sociodemographic | Binary | Male / Female | Male |
| Occupation | Sociodemographic | Categorical (6–level) | 1. Professional (reference) 2. Clerical and service worker 3. Production worker 4. Student 5. Homemaker/Retired/Not employed nor schooling 6. Other | Professional |
| Annual personal income (past 12 months) | Sociodemographic | Categorical (7–level) | 1. < 10,000 CNY  2. 10,000–49,999 CNY  3. 50,000–99,999 CNY  4. 100,000–199,999 CNY  5. 200,000–499,999 CNY  6. ≥ 500,000 CNY  7. Not sure/Don’t know | < 10,000 CNY |
| Residential area | Sociodemographic | Categorical (3–level) | 1. City center  2. Suburban  3. Outer suburban and Other | City center |
| Length of residence in current city | Sociodemographic | Ordinal | 1. 0.5–1 year  2. 1–2 years  3. 2–3 years  4. 3–5 years  5. ≥ 5 years | ≥5 years |
| Living arrangement | Sociodemographic | Binary | Living alone / Not living alone | Not living alone |
| Chronic medical condition | Health | Binary | No / Yes | No |
| Self–rated health (past 6 months) | Health | Ordinal | 1. Low (0–3)  2. Moderate (4–7)  3. High (8–10) | High |
| COVID–19 vaccination status | Health | Binary | Fully vaccinated / Not fully vaccinated | Fully vaccinated |
| Worry about being infected  How likely do you think you are to be infected in the next respiratory infectious disease season? | Perceived susceptibility | Ordinal | 1. Low: 0–3 2. Moderate: 4–7 3. High: 8–10 | High |
| Perceived knowledge and disease severity  How much you agree or disagree with the following statement?  (1) Influenza and common cold are different. (2) The common cold is a serious disease. | Perceived severity | Binary | Continuous: Mean score of the 2 statements (1–Strongly disagree, 2 –Disagree, 3 –Neutral, 4–Agree, 5–Strongly agree).  Binary: Scores ≤ 3 were classified as low knowledge, and scores >3 as high knowledge. | High knowledge |
| Influenza vaccine confidence  How much you agree or disagree with the following statement?  (1) I worry about serious adverse reactions to vaccines.  (2) New vaccines carry more risk than old vaccines.  (3) People receive too many vaccines; harm outweighs benefit.  (4) The fewer vaccines received in a short time, the better. | Vaccine–related | Binary | Continuous: Mean score of the 4 statements (1–Strongly disagree, 2–Disagree, 3–Neutral, 4–Agree, 5–Strongly agree).  Binary: < 3 high confidence,≥ 3 low confidence. | High |
| Perceived vaccine benefit and necessity  How much you agree or disagree with the following statement?  Vaccines are important for protecting my health. Vaccines are effective. Influenza vaccine only prevents influenza (reverse coded)). | Vaccine–related | Binary | Continuous: Mean score of the 3 statements (1 Strongly disagree, 2 Disagree, 3 Neutral, 4 Agree, 5 Strongly agree).  Binary: Scores ≤ 3 were classified as low benefit, and scores > 3 as high benefit. | High benefit |
| Trust in health authorities   How much you agree or disagree with the following statement?  Government recommends influenza vaccination. I believe vaccines provided by community programs are beneficial. I trust the information I receive about vaccination. I can discuss my vaccination concerns with my doctor. | Vaccine–related | Binary | Continuous: Mean score of the 4 statements (1 Strongly disagree, 2 Disagree, 3 Neutral, 4 Agree, 5 Strongly agree).  Binary: Scores ≤ 3 were classified as low trust, and scores > 3 as high trust. | High trust |
| Preference for infection–derived immunity  How much you agree or disagree with the following statement?  Immunity from infection is better than from vaccination. | Vaccine–related |  | Ordinal: 1 Strongly disagree, 2 Disagree, 3 Half–half/ neutral, 4 Agree, 5 Strongly agree  Binary: 1–3 Low (disagree / neutral), 4–5 High (agree) | High |
| Trust in government’s preventive capacity  How much you agree or disagree with the following statement?  I believe that the government of our country can effectively control infectious diseases. | Perceived external control | Binary | Ordinal: 1 Strongly dis agree, 2 Disagree, 3 Half–half/ neutral, 4 Agree, 5 Strongly agree  Binary: 1–3 low trust in government, 4–5 high trust in government | High trust |

**Table S3. Grouping of Occupational Categories into a Seven–Level Variable**

| **Original Category** | **Grouped Category (6–level)** |
| --- | --- |
| Medical–related professionals (including healthcare workers, medical researchers, hospital/clinic/laboratory staff, and medical students) | Professional (reference) |
| Public transportation workers (drivers, attendants, couriers/delivery workers) | Clerical and service worker |
| Workers in densely populated settings (teachers, education staff, retail, or catering workers) | Clerical and service worker |
| Office workers (indoor settings, in contact with >10 people per day) | Clerical and service worker |
| Factory/construction workers (semi–enclosed environment, moderate contact density) | Production worker |
| Agricultural/outdoor workers (open environment, contact with <5 people per day) | Production worker |
| Home–based/remote workers (no regular outside contact) | Retired/not employed nor schooling/homemakers |
| Cross–border workers (e.g., cross–border drivers, flight attendants) | Clerical and service worker |
| Temporary/freelance workers (no fixed workplace) | Production worker |
| Students (non–medical majors) | Student |
| Nannies/home caregivers | Clerical and service worker |
| Retired persons/homemakers | Retired/not employed nor schooling/homemakers |
| Other (please specify) | Other |

Table S4. Overall and city–specific self–reported influenza vaccine coverage

| City name | Number of vaccinated | Total number | Vaccine coverage (%) |
| --- | --- | --- | --- |
| Beijing | 335 | 1285 | 27.6 |
| Shanghai | 164 | 1131 | 14.5 |
| Guangzhou | 391 | 1236 | 31.6 |
| Shenzhen | 329 | 1184 | 27.8 |
| Tianjin | 450 | 1265 | 35.6 |
| Chongqing | 356 | 1222 | 29.1 |
| Chengdu | 528 | 1366 | 38.7 |
| Overall | 2573 | 8689 | 29.6 |

Predicted proportion of vaccine hesitancy and pre–season influenza vaccine uptake by city and by age group

**We used binary logistic regression model to get the predicted proportion of vaccine hesitancy and pre–season influenza vaccine uptake by city and age group.** The models included city, age, sex, and occupation as covariates. The results were as Table S5.

Table S5. Predicted proportion of vaccine hesitancy and pre–season influenza vaccine uptake by city and by age group.

| **City name** | **Age group** | **Predicted value (95% *CI*)** | |
| --- | --- | --- | --- |
|  |  | **Predicted proportion of influenza vaccine hesitancy** | **Predicted pre–season influenza vaccination coverage** |
| Beijing | Young adults (18–44 years) | 50.3% (47.0––53.7%) | 25.7% (22.9–28.6%) |
|  | Adults (45–64 years) | 44.0% (40.6–47.6%) | 28.5% (25.6–31.7%) |
|  | Older adults (≥ 65 years) | 45.7% (41.1–50.4%) | 38.9% (34.2–43.7%) |
|  | Overall | 46.7% (43.5–49.9%) | 30.8% (27.9–33.8%) |
| Shanghai | Young adults (18–44 years) | 55.9% (52.4–59.4%) | 13.4% (11.3–15.8%) |
|  | Adults (45–64 years) | 49.6% (46.0–53.2%) | 15.2% (12.8–17.9%) |
|  | Older adults (≥ 65 years) | 51.3% (46.7–55.9%) | 22.2% (18.6–26.2%) |
|  | Overall | 52.3% (49.0–55.5%) | 16.6% (14.3–19.2%) |
| Guangzhou | Young adults (18–44 years) | 43.8% (40.5–47.1%) | 26.8% (24.1–29.8%) |
|  | Adults (45–64 years) | 37.7% (34.2–41.3%) | 29.8% (26.5–33.3%) |
|  | Older adults (≥ 65 years) | 39.3% (34.7–44.1%) | 40.3% (35.3–45.5%) |
|  | Overall | 40.2% (37.0–43.5%) | 32.1% (29.0–35.3%) |
| Shenzhen | Young adults (18–44 years) | 49.3% (46.0–52.5%) | 26.5% (23.8–29.4%) |
|  | Adults (45–64 years) | 43.0% (39.3–46.7%) | 29.5% (26.1–33.1%) |
|  | Older adults (≥ 65 years) | 44.6% (39.8–49.6%) | 40% (34.9–45.3%) |
|  | Overall | 45.6% (42.2–49.0%) | 31.7% (28.6–35.1%) |
| Tianjin | Young adults (18–44 years) | 44.7% (41.5–48.0%) | 31.5% (28.6–34.5%) |
|  | Adults (45–64 years) | 38.6% (35.3–42.0%) | 34.7% (31.4–38.1%) |
|  | Older adults (≥ 65 years) | 40.2% (35.8–44.7%) | 45.8% (41–50.7%) |
|  | Overall | 41.1% (38.1–44.2%) | 37.2% (34.2–40.3%) |
| Chongqing | Young adults (18–44 years) | 52.0% (48.8–55.3%) | 26.6% (23.9–29.5%) |
|  | Adults (45–64 years) | 45.7% (42.2–49.3%) | 29.5% (26.3–32.9%) |
|  | Older adults (≥ 65 years) | 47.4% (42.7–52.2%) | 40.0% (35.2–45.0%) |
|  | Overall | 48.4% (45.2–51.6%) | 31.8% (28.8–34.9%) |
| Chengdu | Young adults (18–44 years) | 44.7% (41.6–47.9%) | 37.1% (34.2–40.2%) |
|  | Adults (45–64 years) | 38.6% (35.2–42.1%) | 40.6% (37.0–44.3%) |
|  | Older adults (≥ 65 years) | 40.2% (35.7–44.9%) | 52.1% (47.1–57.1%) |
|  | Overall | 41.2% (38.1–44.3%) | 43.2% (40–46.4%) |

Crude influenza vaccination coverage

Crude influenza vaccination coverage was calculated as the proportion of recruited residents who reported receiving pre–season (2024) influenza vaccine. For each group (overall population or subgroup), coverage (%) was computed using the formula:

$$\text{Coverage (\%)}=\frac{\text{Number of vaccinated individuals}}{\text{Total number of residents in the subgroup}}\times100$$

The **CIs** for each proportion were estimated using the normal approximation method:

$$CI=p\pm1.96\sqrt{\frac{p(1-p)}{n}}$$

where $p$ was the observed vaccination proportion and $n$ is the total number of residents in the group. This method provided an estimate of the precision of the crude vaccination coverage for both the overall population and subgroup analyses.

The following table provided the **crude (unadjusted) influenza coverage**.

Table S6. Crude influenza vaccination coverage by sex, by age, and by city.

|  |  | Beijing | | Shanghai | | Shenzhen | | Guangzhou | | Tianjin | | Chongqing | | Chengdu | |
| --- | --- | --- | --- | --- | --- | --- | --- | --- | --- | --- | --- | --- | --- | --- | --- |
|  | Age group | Vaccinated/Total | Vaccination coverage (95% *CI*) | Vaccinated/Total | Vaccination coverage (95% *CI*) | Vaccinated/Total | Vaccination coverage (95% *CI*) | Vaccinated/Total | Vaccination coverage (95% *CI*) | Vaccinated/Total | Vaccination coverage (95% *CI*) | Vaccinated/Total | Vaccination coverage (95% *CI*) | Vaccinated/Total | Vaccination coverage (95% *CI*) |
| Male | 18–24 years | 12/32 | 37.5 (20.73, 54.27) | 6/43 | 13.95 (3.6, 24.31) | 9/22 | 40.91(20.36, 61.45) | 19/56 | 33.93(21.53, 46.33) | 28/59 | 47.46(34.72, 60.2) | 16/36 | 44.44(28.21, 60.68) | 14/34 | 41.18(24.63, 57.72) |
|  | 25–34 years | 52/138 | 37.68 (29.6, 45.77) | 13/64 | 20.31 (10.46, 30.17) | 39/185 | 21.08(15.2, 26.96) | 48/175 | 27.43(20.82, 34.04) | 28/109 | 25.69(17.49, 33.89) | 41/156 | 26.28(19.37, 33.19) | 51/158 | 32.28(24.99, 39.57) |
|  | 35–44 years | 39/134 | 29.1 (21.41, 36.8) | 10/98 | 10.2 (4.21, 16.2) | 31/132 | 23.48(16.25, 30.72) | 60/173 | 34.68(27.59, 41.77) | 41/130 | 31.54(23.55, 39.53) | 34/136 | 25(17.72, 32.28) | 39/117 | 33.33(24.79, 41.88) |
|  | 45–64 years | 35/212 | 16.51 (11.51, 21.51) | 37/217 | 17.05 (12.05, 22.05) | 20/91 | 21.98(13.47, 30.49) | 55/190 | 28.95(22.5, 35.4) | 69/201 | 34.33(27.76, 40.89) | 50/188 | 26.6(20.28, 32.91) | 82/226 | 36.28(30.01, 42.55) |
|  | 65–74 years | 29/64 | 45.31 (33.12, 57.51) | 13/119 | 10.92(5.32, 16.53) | 16/30 | 53.33(35.48, 71.19) | 12/39 | 30.77(16.28, 45.25) | 20/71 | 28.17(17.71, 38.63) | 21/75 | 28(17.84, 38.16) | 22/50 | 44(30.24, 57.76) |
|  | ≥ 75 years | 11/22 | 50 (29.11, 70.89) | 1/27 | 3.7(–3.42, 10.83) | 7/8 | 87.5(64.58, 110.42) | 45794 | 29.41(7.75, 51.07) | 20/43 | 46.51(31.6, 61.42) | 11/18 | 61.11(38.59, 83.63) | 14/33 | 42.42(25.56, 59.29) |
| Female | 18–24 years | 10/39 | 25.64(11.94, 39.35) | 7/29 | 24.14(8.56, 39.71) | 6/19 | 31.58(10.68, 52.48) | 12/39 | 30.77(16.28, 45.25) | 12/58 | 20.69(10.26, 31.11) | 14/27 | 51.85(33, 70.7) | 18/52 | 34.62(21.68, 47.55) |
|  | 25–34 years | 46/167 | 27.54(20.77, 34.32) | 10/52 | 19.23(8.52, 29.94) | 74/280 | 26.43(21.26, 31.59) | 49/169 | 28.99(22.15, 35.84) | 56/127 | 44.09(35.46, 52.73) | 49/175 | 28(21.35, 34.65) | 90/256 | 35.16(29.31, 41.01) |
|  | 35–44 years | 46/154 | 29.87(22.64, 37.1) | 21/140 | 15(9.09, 20.91) | 60/230 | 26.09(20.41, 31.76) | 51/144 | 35.42(27.61, 43.23) | 59/150 | 39.33(31.52, 47.15) | 32/142 | 22.54(15.66, 29.41) | 68/155 | 43.87(36.06, 51.68) |
|  | 45–64 years | 44/221 | 19.91(14.64, 25.17) | 28/207 | 13.53(8.87, 18.19) | 48/149 | 32.21(24.71, 39.72) | 58/173 | 33.53(26.49, 40.56) | 83/216 | 38.43(31.94, 44.91) | 59/177 | 33.33(26.39, 40.28) | 82/194 | 42.27(35.32, 49.22) |
|  | 65–74 years | 24/80 | 30(19.96, 40.04) | 13/99 | 13.13(6.48, 19.78) | 15/31 | 48.39(30.79, 65.98) | 16/48 | 33.33(20, 46.67) | 24/72 | 33.33(22.44, 44.22) | 23/77 | 29.87(19.65, 40.09) | 39/74 | 52.7(41.33, 64.08) |
|  | ≥ 75 years | 7/22 | 31.82(12.35, 51.28) | 5/36 | 13.89(2.59, 25.19) | 4/7 | 57.14(20.48, 93.8) | 6/13 | 46.15(19.05, 73.25) | 10/29 | 34.48(17.18, 51.78) | 6/15 | 40(15.21, 64.79) | 9/17 | 52.94(29.21, 76.67) |
|  | Overall | 355/1285 | 27.63(25.18, 30.07) | 164/1131 | 14.50(12.45, 16.55) | 329/1184 | 27.79(25.24, 30.34) | 391/1236 | 31.63(29.04, 34.23) | 450/1265 | 35.57(32.93, 38.21) | 356/1222 | 29.13(26.58, 31.68) | 528/1366 | 38.65(36.07, 41.24) |
| **Overall** |  | **2573/8689** | **29.61(28.65, 30.57)** |  |  |  |  |  |  |  |  |  |  |  |  |

Table S7. Demographic characteristics and potential factors associated with influenza vaccine hesitancy across seven Chinese megacities

| Characteristics | Beijing | | | Shanghai | | | Guangzhou | | | Shenzhen | | | Tianjin | | | Chongqing | | | Chengdu | | |
| --- | --- | --- | --- | --- | --- | --- | --- | --- | --- | --- | --- | --- | --- | --- | --- | --- | --- | --- | --- | --- | --- |
|  | No/low hesitancy N = 677 | Hesitancy N = 608 | *P*–value | No/low hesitancy  N = 509 | Hesitancy N = 622 | *P*–value | No/low hesitancy N = 781 | Hesitancy N = 455 | *P*–value | No/low hesitancy N = 637 | Hesitancy N = 547 | *P*–value | No/low hesitancy N = 755 | Hesitancy N = 510 | *P*–value | No/low hesitancy N = 625 | Hesitancy N = 597 | *P*–value | No/low hesitancy N = 777 | Hesitancy N = 589 | *P*–value |
| Sex |  |  | 0.90 |  |  | 0.15 |  |  | <0.01 |  |  | <0.01 |  |  | 0.20 |  |  | <0.01 |  |  | 0.08 |
| Male | 317 (47%) | 285 (47%) |  | 243 (48%) | 325 (52%) |  | 384 (49%) | 266 (58%) |  | 222 (35%) | 246 (45%) |  | 353 (47%) | 260 (51%) |  | 276 (44%) | 333 (56%) |  | 335 (43%) | 283 (48%) |  |
| Female | 360 (53%) | 323 (53%) |  | 266 (52%) | 297 (48%) |  | 397 (51%) | 189 (42%) |  | 415 (65%) | 301 (55%) |  | 402 (53%) | 250 (49%) |  | 349 (56%) | 264 (44%) |  | 442 (57%) | 306 (52%) |  |
| Age | 44 ± 15 | 45 ± 16 | 0.20 | 51 ± 15 | 51 ± 17 | 0.60 | 41 ± 14 | 43 ± 16 | 0.05 | 41 ± 14 | 37 ± 11 | <0.01 | 46 ± 17 | 47 ± 17 | 0.20 | 43 ± 15 | 45 ± 16 | 0.02 | 43 ± 15 | 44 ± 17 | 0.20 |
| Age group | |  | 0.03 |  |  | 0.01 |  |  | <0.01 |  |  | <0.01 |  |  | 0.20 |  |  | 0.08 |  |  | 0.10 |
| Young adults (18–44 years) | 352 (52%) | 312 (51%) |  | 195 (38%) | 231 (37%) |  | 502 (64%) | 254 (56%) |  | 437 (69%) | 431 (79%) |  | 389 (52%) | 244 (48%) |  | 360 (58%) | 312 (52%) |  | 458 (59%) | 314 (53%) |  |
| Adults (45–64 years) | 242 (36%) | 191 (31%) |  | 208 (41%) | 215 (35%) |  | 218 (28%) | 145 (32%) |  | 138 (22%) | 102 (19%) |  | 250 (33%) | 167 (33%) |  | 183 (29%) | 182 (30%) |  | 228 (29%) | 192 (33%) |  |
| Older adults (≥ 65 years) | 83 (12%) | 105 (17%) |  | 106 (21%) | 176 (28%) |  | 61 (7.8%) | 56 (12%) |  | 62 (9.7%) | 14 (2.6%) |  | 116 (15%) | 99 (19%) |  | 82 (13%) | 103 (17%) |  | 91 (12%) | 83 (14%) |  |
| Type of residence | |  | 0.60 |  |  | 0.40 |  |  | <0.01 |  |  | <0.01 |  |  | 0.50 |  |  | 0.90 |  |  | <0.01 |
| City center | 332 (49%) | 283 (47%) |  | 205 (40%) | 228 (37%) |  | 340 (44%) | 159 (35%) |  | 431 (68%) | 307 (56%) |  | 145 (19%) | 85 (17%) |  | 530 (85%) | 510 (85%) |  | 437 (56%) | 316 (54%) |  |
| Suburban | 265 (39%) | 256 (42%) |  | 157 (31%) | 212 (34%) |  | 348 (45%) | 208 (46%) |  | 181 (28%) | 201 (37%) |  | 270 (36%) | 189 (37%) |  | 82 (13%) | 73 (12%) |  | 310 (40%) | 225 (38%) |  |
| Outer suburban and other | 80 (12%) | 69 (11%) |  | 147 (29%) | 182 (29%) |  | 93 (12%) | 88 (19%) |  | 25 (3.9%) | 39 (7.1%) |  | 340 (45%) | 236 (46%) |  | 13 (2.1%) | 14 (2.3%) |  | 30 (3.9%) | 48 (8.1%) |  |
| Occupation | |  | <0.01 |  |  | 0.03 |  |  | <0.01 |  |  | <0.01 |  |  | <0.01 |  |  | <0.01 |  |  | <0.01 |
| Professional | 139 (21%) | 65 (11%) |  | 31 (6.1%) | 22 (3.5%) |  | 440 (56%) | 122 (27%) |  | 150 (24%) | 67 (12%) |  | 258 (34%) | 107 (21%) |  | 208 (33%) | 76 (13%) |  | 151 (19%) | 74 (13%) |  |
| Clerical and service worker | 321 (47%) | 284 (47%) |  | 235 (46%) | 248 (40%) |  | 159 (20%) | 134 (29%) |  | 255 (40%) | 311 (57%) |  | 181 (24%) | 148 (29%) |  | 191 (31%) | 222 (37%) |  | 313 (40%) | 224 (38%) |  |
| Production worker | 51 (7.5%) | 55 (9.0%) |  | 31 (6.1%) | 57 (9.2%) |  | 59 (7.6%) | 66 (15%) |  | 51 (8.0%) | 34 (6.2%) |  | 84 (11%) | 100 (20%) |  | 77 (12%) | 119 (20%) |  | 120 (15%) | 123 (21%) |  |
| Student | 14 (2.1%) | 17 (2.8%) |  | 22 (4.3%) | 37 (5.9%) |  | 29 (3.7%) | 24 (5.3%) |  | 16 (2.5%) | 14 (2.6%) |  | 56 (7.4%) | 22 (4.3%) |  | 17 (2.7%) | 16 (2.7%) |  | 16 (2.1%) | 17 (2.9%) |  |
| Homemaker/Retired/Not employed nor schooling | 126 (19%) | 151 (25%) |  | 168 (33%) | 227 (36%) |  | 83 (11%) | 98 (22%) |  | 128 (20%) | 80 (15%) |  | 145 (19%) | 117 (23%) |  | 110 (18%) | 129 (22%) |  | 137 (18%) | 116 (20%) |  |
| Other | 26 (3.8%) | 36 (5.9%) |  | 22 (4.3%) | 31 (5.0%) |  | 11 (1.4%) | 11 (2.4%) |  | 37 (5.8%) | 41 (7.5%) |  | 31 (4.1%) | 16 (3.1%) |  | 22 (3.5%) | 35 (5.9%) |  | 40 (5.1%) | 35 (5.9%) |  |
| Annual income | |  | 0.60 |  |  | <0.01 |  |  | <0.01 |  |  | 0.08 |  |  | 0.03 |  |  | 0.20 |  |  | 0.90 |
| <10,000 CNY | 109 (16%) | 106 (17%) |  | 49 (9.6%) | 71 (11%) |  | 122 (16%) | 123 (27%) |  | 89 (14%) | 89 (16%) |  | 176 (23%) | 139 (27%) |  | 102 (16%) | 91 (15%) |  | 143 (18%) | 115 (20%) |  |
| 10,000–49,999 CNY | 158 (23%) | 153 (25%) |  | 112 (22%) | 204 (33%) |  | 173 (22%) | 129 (28%) |  | 150 (24%) | 109 (20%) |  | 208 (28%) | 154 (30%) |  | 224 (36%) | 212 (36%) |  | 293 (38%) | 222 (38%) |  |
| 50,000–99,999 CNY | 174 (26%) | 142 (23%) |  | 170 (33%) | 150 (24%) |  | 165 (21%) | 100 (22%) |  | 125 (20%) | 93 (17%) |  | 184 (24%) | 98 (19%) |  | 182 (29%) | 162 (27%) |  | 192 (25%) | 143 (24%) |  |
| 100,000–199,999 CNY | 161 (24%) | 129 (21%) |  | 125 (25%) | 112 (18%) |  | 213 (27%) | 53 (12%) |  | 117 (18%) | 107 (20%) |  | 116 (15%) | 61 (12%) |  | 80 (13%) | 70 (12%) |  | 57 (7.3%) | 43 (7.3%) |  |
| 200,000–499,999 CNY | 36 (5.3%) | 34 (5.6%) |  | 31 (6.1%) | 36 (5.8%) |  | 74 (9.5%) | 21 (4.6%) |  | 100 (16%) | 80 (15%) |  | 11 (1.5%) | 9 (1.8%) |  | 8 (1.3%) | 9 (1.5%) |  | 23 (3.0%) | 12 (2.0%) |  |
| $\geq$ 500,000 CNY | 11 (1.6%) | 8 (1.3%) |  | 13 (2.6%) | 13 (2.1%) |  | 14 (1.8%) | 5 (1.1%) |  | 20 (3.1%) | 15 (2.7%) |  | 17 (2.3%) | 6 (1.2%) |  | 7 (1.1%) | 11 (1.8%) |  | 14 (1.8%) | 8 (1.4%) |  |
| Unclear/Unknown | 28 (4.1%) | 36 (5.9%) |  | 9 (1.8%) | 36 (5.8%) |  | 20 (2.6%) | 24 (5.3%) |  | 36 (5.7%) | 54 (9.9%) |  | 43 (5.7%) | 43 (8.4%) |  | 22 (3.5%) | 42 (7.0%) |  | 55 (7.1%) | 46 (7.8%) |  |
| Chronic medical condition | | | 0.90 |  |  | 0.20 |  |  | 0.09 |  |  | 0.06 |  |  | 0.90 |  |  | <0.01 |  |  | 0.30 |
| Yes | 131 (19%) | 120 (20%) |  | 139 (27%) | 193 (31%) |  | 116 (15%) | 85 (19%) |  | 88 (14%) | 55 (10%) |  | 158 (21%) | 105 (21%) |  | 95 (15%) | 141 (24%) |  | 111 (14%) | 96 (16%) |  |
| No | 546 (81%) | 488 (80%) |  | 370 (73%) | 429 (69%) |  | 665 (85%) | 370 (81%) |  | 549 (86%) | 492 (90%) |  | 597 (79%) | 405 (79%) |  | 530 (85%) | 456 (76%) |  | 666 (86%) | 493 (84%) |  |
| Fully vaccinated against COVID–19 | | | <0.01 |  |  | 0.01 |  |  | <0.01 |  |  | 0.01 |  |  | 0.30 |  |  | 0.13 |  |  | <0.01 |
| Yes | 633 (94%) | 523 (86%) |  | 451 (89%) | 507 (82%) |  | 727 (93%) | 391 (86%) |  | 595 (93%) | 488 (89%) |  | 678 (90%) | 468 (92%) |  | 594 (95%) | 554 (93%) |  | 735 (95%) | 518 (88%) |  |
| No | 44 (6.5%) | 85 (14%) |  | 58 (11%) | 115 (18%) |  | 54 (6.9%) | 64 (14%) |  | 42 (6.6%) | 59 (11%) |  | 77 (10%) | 42 (8.2%) |  | 31 (5.0%) | 43 (7.2%) |  | 42 (5.4%) | 71 (12%) |  |
| Length of residence | |  | 0.60 |  |  | 0.50 |  |  | <0.01 |  |  | 0.20 |  |  | 0.02 |  |  | 0.80 |  |  | <0.01 |
| 0.5–1 year | 566 (84%) | 517 (85%) |  | 462 (91%) | 545 (88%) |  | 620 (79%) | 301 (66%) |  | 463 (73%) | 383 (70%) |  | 595 (79%) | 406 (80%) |  | 500 (80%) | 483 (81%) |  | 599 (77%) | 409 (69%) |  |
| 1–2 year | 16 (2.4%) | 14 (2.3%) |  | 6 (1.2%) | 12 (1.9%) |  | 22 (2.8%) | 30 (6.6%) |  | 26 (4.1%) | 17 (3.1%) |  | 23 (3.0%) | 20 (3.9%) |  | 23 (3.7%) | 20 (3.4%) |  | 23 (3.0%) | 32 (5.4%) |  |
| 2–3 year | 24 (3.5%) | 27 (4.4%) |  | 20 (3.9%) | 30 (4.8%) |  | 39 (5.0%) | 32 (7.0%) |  | 39 (6.1%) | 48 (8.8%) |  | 31 (4.1%) | 25 (4.9%) |  | 34 (5.4%) | 27 (4.5%) |  | 49 (6.3%) | 30 (5.1%) |  |
| 3–5 year | 38 (5.6%) | 27 (4.4%) |  | 11 (2.2%) | 17 (2.7%) |  | 60 (7.7%) | 57 (13%) |  | 46 (7.2%) | 51 (9.3%) |  | 37 (4.9%) | 35 (6.9%) |  | 40 (6.4%) | 33 (5.5%) |  | 54 (6.9%) | 71 (12%) |  |
| ≥5 year | 33 (4.9%) | 23 (3.8%) |  | 10 (2.0%) | 18 (2.9%) |  | 40 (5.1%) | 35 (7.7%) |  | 63 (9.9%) | 48 (8.8%) |  | 69 (9.1%) | 24 (4.7%) |  | 28 (4.5%) | 34 (5.7%) |  | 52 (6.7%) | 47 (8.0%) |  |
| Living status | |  | 0.40 |  |  | 0.50 |  |  | 0.04 |  |  | 0.30 |  |  | 0.60 |  |  | 0.20 |  |  | 0.70 |
| Alone | 18 (2.7%) | 22 (3.6%) |  | 83 (16%) | 111 (18%) |  | 73 (9.3%) | 27 (5.9%) |  | 34 (5.3%) | 38 (6.9%) |  | 34 (4.5%) | 19 (3.7%) |  | 44 (7.0%) | 30 (5.0%) |  | 31 (4.0%) | 27 (4.6%) |  |
| With others | 659 (97%) | 586 (96%) |  | 426 (84%) | 511 (82%) |  | 708 (91%) | 428 (94%) |  | 603 (95%) | 509 (93%) |  | 721 (95%) | 491 (96%) |  | 581 (93%) | 567 (95%) |  | 746 (96%) | 562 (95%) |  |
| Self–rated health score | 8.25 ± 1.70 | 7.77 ± 1.88 | <0.01 | 7.78 ± 1.64 | 7.29 ± 1.85 | <0.01 | 7.54 ± 1.82 | 7.15 ± 1.94 | <0.01 | 7.93 ± 1.66 | 7.53 ± 1.89 | <0.01 | 8.07 ± 1.79 | 7.44 ± 1.96 | <0.01 | 8.09 ± 1.74 | 7.50 ± 1.88 | <0.01 | 8.15 ± 1.73 | 7.63 ± 1.87 | <0.01 |
| Self–rated health | |  | <0.01 |  |  | <0.01 |  |  | <0.01 |  |  | 0.01 |  |  | <0.01 |  |  | <0.01 |  |  | <0.01 |
| Poor | 12 (1.8%) | 12 (2.0%) |  | 5 (1.0%) | 19 (3.1%) |  | 19 (2.4%) | 16 (3.5%) |  | 6 (0.9%) | 16 (2.9%) |  | 9 (1.2%) | 13 (2.5%) |  | 8 (1.3%) | 10 (1.7%) |  | 9 (1.2%) | 14 (2.4%) |  |
| Fair | 180 (27%) | 221 (36%) |  | 203 (40%) | 291 (47%) |  | 326 (42%) | 234 (51%) |  | 235 (37%) | 227 (41%) |  | 237 (31%) | 230 (45%) |  | 196 (31%) | 271 (45%) |  | 247 (32%) | 246 (42%) |  |
| Good | 485 (72%) | 375 (62%) |  | 301 (59%) | 312 (50%) |  | 436 (56%) | 205 (45%) |  | 396 (62%) | 304 (56%) |  | 509 (67%) | 267 (52%) |  | 421 (67%) | 316 (53%) |  | 521 (67%) | 329 (56%) |  |
| Fully COVID–19 vaccinated^#^ | | | <0.01 |  |  | <0.01 |  |  | <0.01 |  |  | 0.01 |  |  | 0.30 |  |  | 0.13 |  |  | <0.01 |
| Yes | 633 (94%) | 523 (86%) |  | 451 (89%) | 507 (82%) |  | 727 (93%) | 391 (86%) |  | 595 (93%) | 488 (89%) |  | 678 (90%) | 468 (92%) |  | 594 (95%) | 554 (93%) |  | 735 (95%) | 518 (88%) |  |
| No | 44 (6.5%) | 85 (14%) |  | 58 (11%) | 115 (18%) |  | 54 (6.9%) | 64 (14%) |  | 42 (6.6%) | 59 (11%) |  | 77 (10%) | 42 (8.2%) |  | 31 (5.0%) | 43 (7.2%) |  | 42 (5.4%) | 71 (12%) |  |
| Perceived infection risk | | | <0.01 |  |  | <0.01 |  |  | <0.01 |  |  | <0.01 |  |  | <0.01 |  |  | <0.01 |  |  | <0.01 |
| Low | 93 (14%) | 91 (15%) |  | 54 (11%) | 83 (13%) |  | 111 (14%) | 60 (13%) |  | 95 (15%) | 82 (15%) |  | 89 (12%) | 74 (15%) |  | 86 (14%) | 69 (12%) |  | 118 (15%) | 109 (19%) |  |
| Moderate | 271 (40%) | 355 (58%) |  | 247 (49%) | 375 (60%) |  | 415 (53%) | 306 (67%) |  | 308 (48%) | 339 (62%) |  | 347 (46%) | 302 (59%) |  | 297 (48%) | 423 (71%) |  | 351 (45%) | 353 (60%) |  |
| High | 313 (46%) | 162 (27%) |  | 208 (41%) | 164 (26%) |  | 255 (33%) | 89 (20%) |  | 234 (37%) | 126 (23%) |  | 319 (42%) | 134 (26%) |  | 242 (39%) | 105 (18%) |  | 308 (40%) | 127 (22%) |  |
| Knowledge of influenza vaccine | | | <0.01 |  |  | <0.01 |  |  | <0.01 |  |  | <0.01 |  |  | <0.01 |  |  | <0.01 |  |  | <0.01 |
| High | 615 (91%) | 230 (38%) |  | 476 (94%) | 320 (51%) |  | 661 (85%) | 147 (32%) |  | 535 (84%) | 269 (49%) |  | 653 (86%) | 160 (31%) |  | 530 (85%) | 205 (34%) |  | 641 (82%) | 196 (33%) |  |
| Low | 62 (9.2%) | 378 (62%) |  | 33 (6.5%) | 302 (49%) |  | 120 (15%) | 308 (68%) |  | 102 (16%) | 278 (51%) |  | 102 (14%) | 350 (69%) |  | 95 (15%) | 392 (66%) |  | 136 (18%) | 393 (67%) |  |
| Confidence in influenza vaccine | | | <0.01 |  |  | 0.08 |  |  | <0.01 |  |  | <0.01 |  |  | <0.01 |  |  | 0.001 |  |  | 0.091 |
| High | 114 (17%) | 48 (7.9%) |  | 77 (15%) | 71 (11%) |  | 309 (40%) | 80 (18%) |  | 153 (24%) | 89 (16%) |  | 165 (22%) | 66 (13%) |  | 116 (19%) | 70 (12%) |  | 137 (18%) | 83 (14%) |  |
| Low | 563 (83%) | 560 (92%) |  | 432 (85%) | 551 (89%) |  | 472 (60%) | 375 (82%) |  | 484 (76%) | 458 (84%) |  | 590 (78%) | 444 (87%) |  | 509 (81%) | 527 (88%) |  | 640 (82%) | 506 (86%) |  |
| Influenza vaccine benefit | | | <0.01 |  |  | <0.01 |  |  | <0.01 |  |  | <0.01 |  |  | <0.01 |  |  | <0.01 |  |  | <0.01 |
| High | 670 (99%) | 226 (37%) |  | 499 (98%) | 301 (48%) |  | 764 (98%) | 191 (42%) |  | 629 (99%) | 298 (54%) |  | 746 (99%) | 173 (34%) |  | 615 (98%) | 222 (37%) |  | 746 (96%) | 195 (33%) |  |
| Low | 7 (1.0%) | 382 (63%) |  | 10 (2.0%) | 321 (52%) |  | 17 (2.2%) | 264 (58%) |  | 8 (1.3%) | 249 (46%) |  | 9 (1.2%) | 337 (66%) |  | 10 (1.6%) | 375 (63%) |  | 31 (4.0%) | 394 (67%) |  |
| Trust in health authority | |  | <0.01 |  |  | <0.01 |  |  | <0.01 |  |  | <0.01 |  |  | <0.01 |  |  | <0.01 |  |  | <0.01 |
| High | 668 (99%) | 209 (34%) |  | 496 (97%) | 251 (40%) |  | 769 (98%) | 172 (38%) |  | 626 (98%) | 257 (47%) |  | 743 (98%) | 139 (27%) |  | 612 (98%) | 170 (28%) |  | 747 (96%) | 171 (29%) |  |
| Low | 9 (1.3%) | 399 (66%) |  | 13 (2.6%) | 371 (60%) |  | 12 (1.5%) | 283 (62%) |  | 11 (1.7%) | 290 (53%) |  | 12 (1.6%) | 371 (73%) |  | 13 (2.1%) | 427 (72%) |  | 30 (3.9%) | 418 (71%) |  |
| Preference for infection–derived immunity | | | <0.01 |  |  | <0.01 |  |  | <0.01 |  |  | <0.01 |  |  | <0.01 |  |  | <0.01 |  |  | <0.01 |
| High | 416 (61%) | 92 (15%) |  | 292 (57%) | 142 (23%) |  | 290 (37%) | 74 (16%) |  | 291 (46%) | 93 (17%) |  | 371 (49%) | 67 (13%) |  | 285 (46%) | 67 (11%) |  | 387 (50%) | 82 (14%) |  |
| Low | 261 (39%) | 516 (85%) |  | 217 (43%) | 480 (77%) |  | 491 (63%) | 381 (84%) |  | 346 (54%) | 454 (83%) |  | 384 (51%) | 443 (87%) |  | 340 (54%) | 530 (89%) |  | 390 (50%) | 507 (86%) |  |
| Trust in the government’s preventive capacity | | | <0.01 |  |  | <0.01 |  |  | <0.01 |  |  | <0.01 |  |  | <0.01 |  |  | <0.01 |  |  | <0.01 |
| High | 620 (92%) | 214 (35%) | <0.01 | 459 (90%) | 301 (48%) |  | 683 (87%) | 172 (38%) |  | 572 (90%) | 272 (50%) |  | 683 (90%) | 136 (27%) |  | 559 (89%) | 196 (33%) |  | 651 (84%) | 201 (34%) |  |
| Low | 57 (8.4%) | 394 (65%) |  | 50 (9.8%) | 321 (52%) |  | 98 (13%) | 283 (62%) |  | 65 (10%) | 275 (50%) |  | 72 (9.5%) | 374 (73%) |  | 66 (11%) | 401 (67%) |  | 126 (16%) | 388 (66%) |  |

^#^: Full COVID–19 vaccination was defined according to national guidelines as receipt of at least three doses of an inactivated COVID–19 vaccine during pandemic period.

Table S8. Demographics and potential factors associated with influenza uptake across seven Chinese megacities

| Characteristics | Beijing | | | Shanghai | | | Guangzhou | | | Shenzhen | | | Tianjin | | | Chongqing | | | Chengdu | | |
| --- | --- | --- | --- | --- | --- | --- | --- | --- | --- | --- | --- | --- | --- | --- | --- | --- | --- | --- | --- | --- | --- |
|  | Not vaccinated (N = 930) | Vaccinated  (N = 355) | *P*–value | Not vaccinated  (N = 967) | Vaccinated  (N = 164) | *P*–value | Not vaccinated  (N = 845) | Vaccinated (N = 391) | *P*–value | Not vaccinated (N = 855) | Vaccinated (N = 329) | *P*–value | Not vaccinated (N = 815) | Vaccinated (N = 450) | *P*–value | Not vaccinated (N = 866) | Vaccinated (N = 356) | *P*–value | Not vaccinated (N = 838) | Vaccinated (N = 528) | *P*–value |
| Sex |  |  | 0.20 |  |  | 0.80 |  |  | 0.50 |  |  | 0.30 |  |  | 0.20 |  |  | 0.60 |  |  | 0.07 |
| Male | 424 (46%) | 178 (50%) |  | 488 (50%) | 80 (49%) |  | 451 (53%) | 199 (51%) |  | 346 (40%) | 122 (37%) |  | 407 (50%) | 206 (46%) |  | 436 (50%) | 173 (49%) |  | 396 (47%) | 222 (42%) |  |
| Female | 506 (54%) | 177 (50%) |  | 479 (50%) | 84 (51%) |  | 394 (47%) | 192 (49%) |  | 509 (60%) | 207 (63%) |  | 408 (50%) | 244 (54%) |  | 430 (50%) | 183 (51%) |  | 442 (53%) | 306 (58%) |  |
| Age | 45 ± 15 | 45 ± 17 | 0.80 | 51 ± 16 | 49 ± 16 | 0.11 | 42 ± 14 | 42 ± 14 | 0.90 | 39 ± 12 | 42 ± 15 | <0.01 | 47 ± 17 | 46 ± 17 | 0.60 | 44 ± 15 | 45 ± 17 | 0.50 | 42 ± 16 | 45 ± 16 | 0.01 |
| Age group |  |  | <0.01 |  |  | 0.20 |  |  | 0.90 |  |  | <0.01 |  |  | 0.90 |  |  | 0.30 |  |  | 0.01 |
| Young adults (18–44 years) | 459 (49%) | 205 (58%) |  | 359 (37%) | 67 (41%) |  | 517 (61%) | 239 (61%) |  | 649 (76%) | 219 (67%) |  | 409 (50%) | 224 (50%) |  | 486 (56%) | 186 (52%) |  | 492 (59%) | 280 (53%) |  |
| Adults (45–64 years) | 354 (38%) | 79 (22%) |  | 358 (37%) | 65 (40%) |  | 250 (30%) | 113 (29%) |  | 172 (20%) | 68 (21%) |  | 265 (33%) | 152 (34%) |  | 256 (30%) | 109 (31%) |  | 256 (31%) | 164 (31%) |  |
| Older adults (≥ 65 years) | 117 (13%) | 71 (20%) |  | 250 (26%) | 32 (20%) |  | 78 (9.2%) | 39 (10.0%) | | 34 (4.0%) | 42 (13%) |  | 141 (17%) | 74 (16%) |  | 124 (14%) | 61 (17%) |  | 90 (11%) | 84 (16%) |  |
| Type of residence | |  | 0.90 |  |  | 0.09 |  |  | 0.04 |  |  | 0.01 |  |  | <0.01 |  |  | 0.80 |  |  | 0.90 |
| City center | 444 (48%) | 171 (48%) |  | 373 (39%) | 60 (37%) |  | 322 (38%) | 177 (45%) |  | 510 (60%) | 228 (69%) |  | 155 (19%) | 75 (17%) |  | 741 (86%) | 299 (84%) |  | 463 (55%) | 290 (55%) |  |
| Suburban | 376 (40%) | 145 (41%) |  | 304 (31%) | 65 (40%) |  | 390 (46%) | 166 (42%) |  | 293 (34%) | 89 (27%) |  | 252 (31%) | 207 (46%) |  | 106 (12%) | 49 (14%) |  | 326 (39%) | 209 (40%) |  |
| Outer suburban and other | 110 (12%) | 39 (11%) |  | 290 (30%) | 39 (24%) |  | 133 (16%) | 48 (12%) |  | 52 (6.1%) | 12 (3.6%) |  | 408 (50%) | 168 (37%) |  | 19 (2.2%) | 8 (2.2%) |  | 49 (5.8%) | 29 (5.5%) |  |
| Occupation |  |  | <0.01 |  |  | 0.12 |  |  | 0.70 |  |  | <0.01 |  |  | <0.01 |  |  | <0.01 |  |  | 0.11 |
| Professional | 128 (14%) | 76 (21%) |  | 40 (4.1%) | 13 (7.9%) |  | 379 (45%) | 183 (47%) |  | 125 (15%) | 92 (28%) |  | 194 (24%) | 171 (38%) |  | 171 (20%) | 113 (32%) |  | 141 (17%) | 84 (16%) |  |
| Clerical and service worker | 456 (49%) | 149 (42%) |  | 409 (42%) | 74 (45%) |  | 196 (23%) | 97 (25%) |  | 449 (53%) | 117 (36%) |  | 223 (27%) | 106 (24%) |  | 322 (37%) | 91 (26%) |  | 330 (39%) | 207 (39%) |  |
| Production worker | 83 (8.9%) | 23 (6.5%) |  | 76 (7.9%) | 12 (7.3%) |  | 91 (11%) | 34 (8.7%) |  | 61 (7.1%) | 24 (7.3%) |  | 121 (15%) | 63 (14%) |  | 148 (17%) | 48 (13%) |  | 149 (18%) | 94 (18%) |  |
| Student | 18 (1.9%) | 13 (3.7%) |  | 49 (5.1%) | 10 (6.1%) |  | 35 (4.1%) | 18 (4.6%) |  | 21 (2.5%) | 9 (2.7%) |  | 54 (6.6%) | 24 (5.3%) |  | 20 (2.3%) | 13 (3.7%) |  | 17 (2.0%) | 16 (3.0%) |  |
| Homemaker/Retired/Not employed nor schooling | 195 (21%) | 82 (23%) |  | 350 (36%) | 45 (27%) |  | 130 (15%) | 51 (13%) |  | 137 (16%) | 71 (22%) |  | 193 (24%) | 69 (15%) |  | 164 (19%) | 75 (21%) |  | 145 (17%) | 108 (20%) |  |
| Other | 50 (5.4%) | 12 (3.4%) |  | 43 (4.4%) | 10 (6.1%) |  | 14 (1.7%) | 8 (2.0%) |  | 62 (7.3%) | 16 (4.9%) |  | 30 (3.7%) | 17 (3.8%) |  | 41 (4.7%) | 16 (4.5%) |  | 56 (6.7%) | 19 (3.6%) |  |
| Annual income | |  | 0.20 |  |  |  |  |  | 0.20 |  |  | 0.50 |  |  | 0.50 |  |  |  |  |  | 0.90 |
| < 10,000 CNY | 165 (18%) | 50 (14%) |  | 97 (10%) | 23 (14%) |  | 168 (20%) | 77 (20%) |  | 123 (14%) | 55 (17%) |  | 208 (26%) | 107 (24%) |  | 135 (16%) | 58 (16%) |  | 158 (19%) | 100 (19%) |  |
| 10,000–49,999 CNY | 225 (24%) | 86 (24%) |  | 286 (30%) | 30 (18%) |  | 214 (25%) | 88 (23%) |  | 178 (21%) | 81 (25%) |  | 243 (30%) | 119 (26%) |  | 318 (37%) | 118 (33%) |  | 308 (37%) | 207 (39%) |  |
| 50,000–99,999 CNY | 233 (25%) | 83 (23%) |  | 280 (29%) | 40 (24%) |  | 185 (22%) | 80 (20%) |  | 157 (18%) | 61 (19%) |  | 175 (21%) | 107 (24%) |  | 246 (28%) | 98 (28%) |  | 205 (24%) | 130 (25%) |  |
| 100,000–199,999 CNY | 206 (22%) | 84 (24%) |  | 201 (21%) | 36 (22%) |  | 174 (21%) | 92 (24%) |  | 168 (20%) | 56 (17%) |  | 113 (14%) | 64 (14%) |  | 99 (11%) | 51 (14%) |  | 64 (7.6%) | 36 (6.8%) |  |
| 200,000–499,999 CNY | 50 (5.4%) | 20 (5.6%) |  | 50 (5.2%) | 17 (10%) |  | 69 (8.2%) | 26 (6.6%) |  | 131 (15%) | 49 (15%) |  | 13 (1.6%) | 7 (1.6%) |  | 11 (1.3%) | 6 (1.7%) |  | 21 (2.5%) | 14 (2.7%) |  |
| $\geq$500,000 CNY | 10 (1.1%) | 9 (2.5%) |  | 17 (1.8%) | 9 (5.5%) |  | 10 (1.2%) | 9 (2.3%) |  | 27 (3.2%) | 8 (2.4%) |  | 11 (1.3%) | 12 (2.7%) |  | 13 (1.5%) | 5 (1.4%) |  | 15 (1.8%) | 7 (1.3%) |  |
| Unclear/Unknown | 41 (4.4%) | 23 (6.5%) |  | 36 (3.7%) | 9 (5.5%) |  | 25 (3.0%) | 19 (4.9%) |  | 71 (8.3%) | 19 (5.8%) |  | 52 (6.4%) | 34 (7.6%) |  | 44 (5.1%) | 20 (5.6%) |  | 67 (8.0%) | 34 (6.4%) |  |
| Chronic medical condition | | | 0.30 |  |  | 0.90 |  |  | 0.20 |  |  | 0.50 |  |  | 0.90 |  |  | 0.04 |  |  | 0.20 |
| Yes | 174 (19%) | 77 (22%) |  | 284 (29%) | 48 (29%) |  | 145 (17%) | 56 (14%) |  | 99 (12%) | 44 (13%) |  | 170 (21%) | 93 (21%) |  | 154 (18%) | 82 (23%) |  | 119 (14%) | 88 (17%) |  |
| No | 756 (81%) | 278 (78%) |  | 683 (71%) | 116 (71%) |  | 700 (83%) | 335 (86%) |  | 756 (88%) | 285 (87%) |  | 645 (79%) | 357 (79%) |  | 712 (82%) | 274 (77%) |  | 719 (86%) | 440 (83%) |  |
| Fully vaccinated against COVID–19 | | | <0.01 |  |  | <0.01 |  |  | <0.01 |  |  | <0.01 |  |  | <0.01 |  |  | 0.11 |  |  | <0.01 |
| Yes | 814 (88%) | 342 (96%) |  | 803 (83%) | 155 (95%) |  | 747 (88%) | 371 (95%) |  | 765 (89%) | 318 (97%) |  | 711 (87%) | 435 (97%) |  | 807 (93%) | 341 (96%) |  | 744 (89%) | 509 (96%) |  |
| No | 116 (12%) | 13 (3.7%) |  | 164 (17%) | 9 (5.5%) |  | 98 (12%) | 20 (5.1%) |  | 90 (11%) | 11 (3.3%) |  | 104 (13%) | 15 (3.3%) |  | 59 (6.8%) | 15 (4.2%) |  | 94 (11%) | 19 (3.6%) |  |
| Length of residence | |  | 0.90 |  |  | 0.40 |  |  | 0.20 |  |  | 0.90 |  |  | <0.01 |  |  | 0.30 |  |  | 0.05 |
| 0.5–1 year | 785 (84%) | 298 (84%) |  | 867 (90%) | 140 (85%) |  | 617 (73%) | 304 (78%) |  | 609 (71%) | 237 (72%) |  | 614 (75%) | 387 (86%) |  | 698 (81%) | 285 (80%) |  | 594 (71%) | 414 (78%) |  |
| 1–2 years | 23 (2.5%) | 7 (2.0%) |  | 16 (1.7%) | 2 (1.2%) |  | 41 (4.9%) | 11 (2.8%) |  | 30 (3.5%) | 13 (4.0%) |  | 28 (3.4%) | 15 (3.3%) |  | 29 (3.3%) | 14 (3.9%) |  | 36 (4.3%) | 19 (3.6%) |  |
| 2–3 years | 37 (4.0%) | 14 (3.9%) |  | 40 (4.1%) | 10 (6.1%) |  | 48 (5.7%) | 23 (5.9%) |  | 62 (7.3%) | 25 (7.6%) |  | 42 (5.2%) | 14 (3.1%) |  | 45 (5.2%) | 16 (4.5%) |  | 55 (6.6%) | 24 (4.5%) |  |
| 3–5 years | 47 (5.1%) | 18 (5.1%) |  | 22 (2.3%) | 6 (3.7%) |  | 81 (9.6%) | 36 (9.2%) |  | 72 (8.4%) | 25 (7.6%) |  | 53 (6.5%) | 19 (4.2%) |  | 56 (6.5%) | 17 (4.8%) |  | 85 (10%) | 40 (7.6%) |  |
| ≥ 5 years | 38 (4.1%) | 18 (5.1%) |  | 22 (2.3%) | 6 (3.7%) |  | 58 (6.9%) | 17 (4.3%) |  | 82 (9.6%) | 29 (8.8%) |  | 78 (9.6%) | 15 (3.3%) |  | 38 (4.4%) | 24 (6.7%) |  | 68 (8.1%) | 31 (5.9%) |  |
| Living status |  |  | 0.60 |  |  | 0.14 |  |  | 0.20 |  |  | 0.90 |  |  | 0.80 |  |  | 0.40 |  |  | 0.20 |
| Alone | 31 (3.3%) | 9 (2.5%) |  | 173 (18%) | 21 (13%) |  | 75 (8.9%) | 25 (6.4%) |  | 53 (6.2%) | 19 (5.8%) |  | 33 (4.0%) | 20 (4.4%) |  | 49 (5.7%) | 25 (7.0%) |  | 41 (4.9%) | 17 (3.2%) |  |
| With others | 899 (97%) | 346 (97%) |  | 794 (82%) | 143 (87%) |  | 770 (91%) | 366 (94%) |  | 802 (94%) | 310 (94%) |  | 782 (96%) | 430 (96%) |  | 817 (94%) | 331 (93%) |  | 797 (95%) | 511 (97%) |  |
| Self–rated health score | 8.04 ± 1.77 | 7.99 ± 1.91 | 0.70 | 7.49 ± 1.77 | 7.65 ± 1.85 | 0.30 | 7.28 ± 1.92 | 7.65 ± 1.75 | <0.01 | 7.70 ± 1.78 | 7.87 ± 1.79 | 0.14 | 7.73 ± 1.84 | 7.98 ± 1.96 | 0.03 | 7.81 ± 1.85 | 7.78 ± 1.80 | 0.80 | 7.94 ± 1.78 | 7.90 ± 1.85 | 0.70 |
| Self–rated health | |  | 0.30 |  |  | 0.90 |  |  | 0.02 |  |  | 0.20 |  |  | 0.50 |  |  | 0.90 |  |  | 0.40 |
| Poor | 14 (1.5%) | 10 (2.8%) |  | 21 (2.2%) | 3 (1.8%) |  | 29 (3.4%) | 6 (1.5%) |  | 16 (1.9%) | 6 (1.8%) |  | 13 (1.6%) | 9 (2.0%) |  | 13 (1.5%) | 5 (1.4%) |  | 17 (2.0%) | 6 (1.1%) |  |
| Fair | 289 (31%) | 112 (32%) |  | 422 (44%) | 72 (44%) |  | 398 (47%) | 162 (41%) |  | 347 (41%) | 115 (35%) |  | 310 (38%) | 157 (35%) |  | 333 (38%) | 134 (38%) |  | 296 (35%) | 197 (37%) |  |
| Good | 627 (67%) | 233 (66%) |  | 524 (54%) | 89 (54%) |  | 418 (49%) | 223 (57%) |  | 492 (58%) | 208 (63%) |  | 492 (60%) | 284 (63%) |  | 520 (60%) | 217 (61%) |  | 525 (63%) | 325 (62%) |  |
| Fully COVID–19 vaccinated^#^ | | | <0.01 |  |  | 0.90 |  |  | <0.01 |  |  | <0.01 |  |  | <0.01 |  |  | 0.11 |  |  | <0.01 |
| Yes | 814 (88%) | 342 (96%) |  | 284 (29%) | 48 (29%) |  | 747 (88%) | 371 (95%) |  | 765 (89%) | 318 (97%) |  | 711 (87%) | 435 (97%) |  | 807 (93%) | 341 (96%) |  | 744 (89%) | 509 (96%) |  |
| No | 116 (12%) | 13 (3.7%) |  | 683 (71%) | 116 (71%) |  | 98 (12%) | 20 (5.1%) |  | 90 (11%) | 11 (3.3%) |  | 104 (13%) | 15 (3.3%) |  | 59 (6.8%) | 15 (4.2%) |  | 94 (11%) | 19 (3.6%) |  |
| Perceived infection risk | | | <0.01 |  |  | 0.50 |  |  | 0.01 |  |  | 0.20 |  |  | 0.05 |  |  | <0.01 |  |  | 0.40 |
| Low | 136 (15%) | 48 (14%) |  | 116 (12%) | 21 (13%) |  | 108 (13%) | 63 (16%) |  | 120 (14%) | 57 (17%) |  | 93 (11%) | 70 (16%) |  | 105 (12%) | 50 (14%) |  | 146 (17%) | 81 (15%) |  |
| Moderate | 476 (51%) | 150 (42%) |  | 539 (56%) | 83 (51%) |  | 518 (61%) | 203 (52%) |  | 480 (56%) | 167 (51%) |  | 415 (51%) | 234 (52%) |  | 538 (62%) | 182 (51%) |  | 435 (52%) | 269 (51%) |  |
| High | 318 (34%) | 157 (44%) |  | 312 (32%) | 60 (37%) |  | 219 (26%) | 125 (32%) |  | 255 (30%) | 105 (32%) |  | 307 (38%) | 146 (32%) |  | 223 (26%) | 124 (35%) |  | 257 (31%) | 178 (34%) |  |
| Knowledge of influenza vaccine | | | <0.01 |  |  | 0.03 |  |  | <0.01 |  |  | <0.01 |  |  | 0.20 |  |  | <0.01 |  |  | <0.01 |
| High | 591 (64%) | 254 (72%) |  | 668 (69%) | 128 (78%) |  | 523 (62%) | 285 (73%) |  | 557 (65%) | 247 (75%) |  | 513 (63%) | 300 (67%) |  | 489 (56%) | 246 (69%) |  | 481 (57%) | 356 (67%) |  |
| Low | 339 (36%) | 101 (28%) |  | 299 (31%) | 36 (22%) |  | 322 (38%) | 106 (27%) |  | 298 (35%) | 82 (25%) |  | 302 (37%) | 150 (33%) |  | 377 (44%) | 110 (31%) |  | 357 (43%) | 172 (33%) |  |
| Confidence in influenza vaccine | | | <0.01 |  |  | <0.01 |  |  | 0.01 |  |  | <0.01 |  |  | 0.02 |  |  | <0.01 |  |  | 0.50 |
| High | 93 (10%) | 69 (19%) |  | 111 (11%) | 37 (23%) |  | 245 (29%) | 144 (37%) |  | 147 (17%) | 95 (29%) |  | 133 (16%) | 98 (22%) |  | 114 (13%) | 72 (20%) |  | 130 (16%) | 90 (17%) |  |
| Low | 837 (90%) | 286 (81%) |  | 856 (89%) | 127 (77%) |  | 600 (71%) | 247 (63%) |  | 708 (83%) | 234 (71%) |  | 682 (84%) | 352 (78%) |  | 752 (87%) | 284 (80%) |  | 708 (84%) | 438 (83%) |  |
| Influenza vaccine benefit | | | <0.01 |  |  | 0.03 |  |  | <0.01 |  |  | <0.01 |  |  | 0.03 |  |  | <0.01 |  |  | <0.01 |
| High | 614 (66%) | 282 (79%) |  | 672 (69%) | 128 (78%) |  | 626 (74%) | 329 (84%) |  | 647 (76%) | 280 (85%) |  | 575 (71%) | 344 (76%) |  | 564 (65%) | 273 (77%) |  | 547 (65%) | 394 (75%) |  |
| Low | 316 (34%) | 73 (21%) |  | 295 (31%) | 36 (22%) |  | 219 (26%) | 62 (16%) |  | 208 (24%) | 49 (15%) |  | 240 (29%) | 106 (24%) |  | 302 (35%) | 83 (23%) |  | 291 (35%) | 134 (25%) |  |
| Trust in health authorities | |  | <0.01 |  |  | <0.01 |  |  | <0.01 |  |  | <0.01 |  |  | 0.01 |  |  | <0.01 |  |  | <0.01 |
| High | 590 (63%) | 287 (81%) |  | 618 (64%) | 129 (79%) |  | 609 (72%) | 332 (85%) |  | 603 (71%) | 280 (85%) |  | 548 (67%) | 334 (74%) |  | 519 (60%) | 263 (74%) |  | 518 (62%) | 400 (76%) |  |
| Low | 340 (37%) | 68 (19%) |  | 349 (36%) | 35 (21%) |  | 236 (28%) | 59 (15%) |  | 252 (29%) | 49 (15%) |  | 267 (33%) | 116 (26%) |  | 347 (40%) | 93 (26%) |  | 320 (38%) | 128 (24%) |  |
| Preference for infection–derived immunity | | | <0.01 |  |  | 0.50 |  |  | 0.07 |  |  | 0.01 |  |  | 0.30 |  |  | <0.01 |  |  | <0.01 |
| High | 345 (37%) | 163 (46%) |  | 367 (38%) | 67 (41%) |  | 235 (28%) | 129 (33%) |  | 259 (30%) | 125 (38%) |  | 274 (34%) | 164 (36%) |  | 218 (25%) | 134 (38%) |  | 257 (31%) | 212 (40%) |  |
| Low | 585 (63%) | 192 (54%) |  | 600 (62%) | 97 (59%) |  | 610 (72%) | 262 (67%) |  | 596 (70%) | 204 (62%) |  | 541 (66%) | 286 (64%) |  | 648 (75%) | 222 (62%) |  | 581 (69%) | 316 (60%) |  |
| Trust in the government’s preventive capacity | | | <0.01 |  |  | 0.09 |  |  | <0.01 |  |  | <0.01 |  |  | 0.02 |  |  | <0.01 |  |  | 0.06 |
| High | 565 (61%) | 269 (76%) |  | 640 (66%) | 120 (73%) |  | 554 (66%) | 301 (77%) |  | 588 (69%) | 256 (78%) |  | 508 (62%) | 311 (69%) |  | 498 (58%) | 257 (72%) |  | 506 (60%) | 346 (66%) |  |
| Low | 365 (39%) | 86 (24%) |  | 327 (34%) | 44 (27%) |  | 291 (34%) | 90 (23%) |  | 267 (31%) | 73 (22%) |  | 307 (38%) | 139 (31%) |  | 368 (42%) | 99 (28%) |  | 332 (40%) | 182 (34%) |  |

^#^: Full COVID–19 vaccination was defined according to national guidelines as receipt of at least three doses of an inactivated COVID–19 vaccine during pandemic period.

Table S9. Adjusted odds ratios for potential factors associated with influenza vaccine hesitancy across seven Chinese megacities

| Variables | Beijing | | Shanghai | | Guangzhou | | Shenzhen | | Tianjin | | Chongqing | | Chengdu | |
| --- | --- | --- | --- | --- | --- | --- | --- | --- | --- | --- | --- | --- | --- | --- |
|  | aOR (95% CI) | *P*–value | aOR (95% CI) | *P*–value | aOR (95% CI) | *P*–value | aOR (95% CI) | *P*–value | aOR (95% CI) | *P*–value | aOR (95% CI) | *P*–value | aOR (95% CI) | *P*–value |
| Sex |  |  |  |  |  |  |  |  |  |  |  |  |  |  |
| Male | Reference |  |  |  |  |  |  |  |  |  |  |  |  |  |
| Female | 0.91 (0.62, 1.33) | 0.62 | 0.59 (0.42, 0.84) | < 0.01 | 0.85 (0.59, 1.23) | 0.39 | 0.57 (0.4, 0.8) | < 0.01 | 0.79 (0.51, 1.21) | 0.28 | 0.79 (0.53, 1.18) | 0.25 | 0.97 (0.66, 1.42) | 0.86 |
| Age group |  |  |  |  |  |  |  |  |  |  |  |  |  |  |
| Young adults (18–44 years) | Reference |  |  |  |  |  |  |  |  |  |  |  |  |  |
| Adults (45–64 years) | 0.97 (0.61, 1.53) | 0.89 | 1.07 (0.68, 1.69) | 0.76 | 0.77 (0.48, 1.23) | 0.27 | 0.58 (0.35, 0.98) | 0.04 | 1.06 (0.66, 1.72) | 0.80 | 1.04 (0.66, 1.63) | 0.88 | 0.67 (0.42, 1.08) | 0.10 |
| Older adults (≥ 65 years) | 0.72 (0.3, 1.7) | 0.45 | 1.6 (0.83, 3.1) | 0.16 | 0.94 (0.45, 1.98) | 0.87 | 0.26 (0.09, 0.73) | 0.01 | 0.52 (0.21, 1.28) | 0.16 | 0.5 (0.24, 1.03) | 0.06 | 0.69 (0.37, 1.29) | 0.24 |
| Residence type |  |  |  |  |  |  |  |  |  |  |  |  |  |  |
| City center | Reference |  |  |  |  |  |  |  |  |  |  |  |  |  |
| Suburban | 1.08 (0.73, 1.61) | 0.70 | 0.98 (0.65, 1.47) | 0.90 | 1.41 (0.94, 2.1) | 0.10 | 1.63 (1.13, 2.33) | p < 0.01 | 0.97 (0.53, 1.77) | 0.93 | 0.96 (0.52, 1.75) | 0.89 | 1.18 (0.82, 1.71) | 0.37 |
| Outer suburban and other | 0.59 (0.3, 1.14) | 0.12 | 1.02 (0.67, 1.58) | 0.91 | 2.96 (1.7, 5.14) | p < 0.01 | 2.92 (1.4, 6.09) | p < 0.01 | 0.99 (0.56, 1.75) | 0.96 | 0.97 (0.34, 2.83) | 0.96 | 3.03 (1.55, 5.91) | < 0.01 |
| Occupation |  |  |  |  |  |  |  |  |  |  |  |  |  |  |
| Professional | Reference |  |  |  |  |  |  |  |  |  |  |  |  |  |
| Clerical and service worker | 1.72 (1.01, 2.93) | 0.04 | 1.43 (0.62, 3.31) | 0.40 | 0.91 (0.54, 1.53) | 0.71 | 3.38 (2.03, 5.62) | < 0.01 | 1.16 (0.64, 2.08) | 0.62 | 3.1 (1.78, 5.38) | < 0.01 | 1.37 (0.83, 2.27) | 0.22 |
| Production worker | 2.31 (0.97, 5.49) | 0.06 | 0.65 (0.2, 2.06) | 0.46 | 1.15 (0.6, 2.19) | 0.68 | 1.23 (0.53, 2.86) | 0.64 | 2.45 (1.3, 4.59) | p < 0.01 | 3.55 (1.75, 7.22) | < 0.01 | 1.44 (0.75, 2.78) | 0.27 |
| Student | 2.94 (0.95, 9.11) | 0.06 | 3.05 (0.9, 10.33) | 0.07 | 0.74 (0.25, 2.23) | 0.59 | 0.92 (0.23, 3.65) | 0.91 | 1.03 (0.33, 3.23) | 0.96 | 4.42 (1.36, 14.37) | 0.01 | 1.69 (0.48, 5.95) | 0.42 |
| Homemaker/Retired/Not employed nor schooling | 1.49 (0.67, 3.28) | 0.32 | 0.82 (0.34, 2.01) | 0.66 | 1.19 (0.63, 2.24) | 0.60 | 1.96 (0.95, 4.04) | 0.07 | 1.15 (0.56, 2.36) | 0.70 | 3.42 (1.71, 6.83) | < 0.01 | 1.65 (0.87, 3.15) | 0.13 |
| Other | 1.04 (0.25, 4.32) | 0.96 | 2.78 (0.95, 8.13) | 0.06 | 1.02 (0.29, 3.54) | 0.98 | 2.25 (0.95, 5.33) | 0.07 | 0.86 (0.3, 2.44) | 0.78 | 2.81 (1.01, 7.77) | 0.05 | 1.53 (0.7, 3.36) | 0.29 |
| Annual income |  |  |  |  |  |  |  |  |  |  |  |  |  |  |
| < 10,000 CNY | Reference |  |  |  |  |  |  |  |  |  |  |  |  |  |
| 10,000 – 49,999 CNY | 1.51 (0.79, 2.89) | 0.21 | 1.56 (0.74, 3.29) | 0.24 | 1.15 (0.64, 2.06) | 0.65 | 0.57 (0.31, 1.03) | 0.06 | 0.93 (0.52, 1.65) | 0.80 | 1.7 (0.87, 3.33) | 0.12 | 0.98 (0.6, 1.62) | 0.95 |
| 50,000 – 99,999 CNY | 1.18 (0.62, 2.23) | 0.62 | 0.93 (0.45, 1.95) | 0.86 | 0.81 (0.42, 1.56) | 0.53 | 0.67 (0.36, 1.23) | 0.20 | 0.6 (0.33, 1.11) | 0.10 | 2.05 (1, 4.18) | 0.05 | 1.01 (0.57, 1.78) | 0.98 |
| 100,000 – 199,999 CNY | 1.34 (0.68, 2.64) | 0.40 | 0.95 (0.44, 2.06) | 0.89 | 0.69 (0.34, 1.39) | 0.30 | 0.73 (0.4, 1.31) | 0.29 | 0.54 (0.24, 1.23) | 0.14 | 2.04 (0.89, 4.71) | 0.09 | 2 (0.95, 4.2) | 0.07 |
| 200,000 – 499,999 CNY | 1.51 (0.59, 3.88) | 0.39 | 1.04 (0.4, 2.76) | 0.93 | 0.85 (0.37, 1.94) | 0.70 | 0.57 (0.3, 1.08) | 0.08 | 0.66 (0.16, 2.7) | 0.57 | 0.95 (0.18, 4.94) | 0.95 | 0.73 (0.22, 2.38) | 0.60 |
| $\geq$ 500,000 CNY | 0.55 (0.11, 2.75) | 0.46 | 0.78 (0.18, 3.42) | 0.74 | 0.46 (0.1, 2.16) | 0.32 | 0.35 (0.13, 0.92) | 0.03 | 0.19 (0.07, 0.47) | < 0.01 | 1.92 (0.23, 15.77) | 0.54 | 1.94 (0.5, 7.52) | 0.34 |
| Unclear/Unknown | 0.9 (0.33, 2.47) | 0.84 | 2.86 (1, 8.18) | 0.05 | 2.37 (0.92, 6.06) | 0.07 | 0.46 (0.2, 1.08) | 0.07 | 1.01 (0.38, 2.69) | 0.98 | 3.99 (1.64, 9.71) | < 0.01 | 0.97 (0.46, 2.07) | 0.95 |
| Chronic Medical condition | |  |  |  |  |  |  |  |  |  |  |  |  |  |
| Yes | Reference |  |  |  |  |  |  |  |  |  |  |  |  |  |
| No | 1.64 (0.89, 3.01) | 0.11 | 0.74 (0.49, 1.11) | 0.15 | 1.04 (0.58, 1.86) | 0.89 | 1.3 (0.67, 2.49) | 0.44 | 1.17 (0.66, 2.09) | 0.59 | 0.71 (0.38, 1.32) | 0.28 | 1.12 (0.62, 2.02) | 0.70 |
| Self–rated health |  |  |  |  |  |  |  |  |  |  |  |  |  |  |
| Poor | Reference |  |  |  |  |  |  |  |  |  |  |  |  |  |
| Fair | 0.53 (0.19, 1.48) | 0.23 | 0.65 (0.17, 2.42) | 0.52 | 0.83 (0.35, 1.93) | 0.66 | 0.39 (0.16, 0.97) | 0.04 | 4.87 (1.65, 14.33) | < 0.01 | 2.04 (0.58, 7.14) | 0.27 | 0.86 (0.27, 2.75) | 0.80 |
| Good | 1.28 (0.69, 2.37) | 0.44 | 1.1 (0.5, 2.42) | 0.81 | 1.28 (0.74, 2.22) | 0.38 | 1.61 (0.93, 2.81) | 0.09 | 0.28 (0.14, 0.55) | < 0.01 | 0.88 (0.4, 1.93) | 0.74 | 1.09 (0.54, 2.19) | 0.82 |
| Living status |  |  |  |  |  |  |  |  |  |  |  |  |  |  |
| Living alone | Reference |  |  |  |  |  |  |  |  |  |  |  |  |  |
| Living with others | 0.9 (0.35, 2.36) | 0.83 | 0.86 (0.54, 1.37) | 0.54 | 0.96 (0.5, 1.83) | 0.89 | 1.13 (0.61, 2.06) | 0.70 | 1.28 (0.37, 4.47) | 0.69 | 1.26 (0.51, 3.12) | 0.62 | 0.8 (0.34, 1.88) | 0.61 |
| Fully vaccinated against COVID–19^#^ | |  |  |  |  |  |  |  |  |  |  |  |  |  |
| Yes | Reference |  |  |  |  |  |  |  |  |  |  |  |  |  |
| No | 1.88 (1.07, 3.32) | 0.03 | 1.51 (0.9, 2.52) | 0.12 | 2.02 (1.03, 3.95) | 0.04 | 1.36 (0.72, 2.54) | 0.34 | 1.01 (0.44, 2.3) | 0.98 | 1.04 (0.41, 2.65) | 0.94 | 2.06 (1.09, 3.87) | 0.03 |
| Perceived infection risk of influenza | |  |  |  |  |  |  |  |  |  |  |  |  |  |
| Low | Reference |  |  |  |  |  |  |  |  |  |  |  |  |  |
| Medium | 0.55 (0.37, 0.82) | < 0.01 | 0.77 (0.52, 1.14) | 0.19 | 0.67 (0.44, 1.02) | 0.06 | 0.64 (0.43, 0.94) | 0.02 | 0.61 (0.4, 0.94) | 0.02 | 0.69 (0.44, 1.09) | 0.11 | 0.94 (0.62, 1.41) | 0.75 |
| High | 0.67 (0.48, 0.92) | 0.01 | 1 (0.74, 1.35) | 1.00 | 0.83 (0.6, 1.15) | 0.25 | 0.84 (0.63, 1.12) | 0.24 | 0.94 (0.66, 1.34) | 0.74 | 0.59 (0.43, 0.82) | < 0.01 | 0.69 (0.51, 0.95) | 0.02 |
| Knowledge of influenza vaccine | |  |  |  |  |  |  |  |  |  |  |  |  |  |
| High | Reference |  |  |  |  |  |  |  |  |  |  |  |  |  |
| Low | 3.2 (1.77, 5.79) | < 0.01 | 1.39 (0.82, 2.35) | 0.22 | 2.97 (1.8, 4.9) | < 0.01 | 1.4 (0.89, 2.18) | 0.14 | 1.4 (0.76, 2.58) | 0.29 | 1.56 (0.91, 2.67) | 0.11 | 1.48 (0.89, 2.44) | 0.13 |
| Vaccine confidence |  |  |  |  |  |  |  |  |  |  |  |  |  |  |
| High | Reference |  |  |  |  |  |  |  |  |  |  |  |  |  |
| Low | 2.75 (1.68, 4.5) | < 0.01 | 3.84 (2.28, 6.46) | < 0.01 | 4.18 (2.77, 6.32) | < 0.01 | 1.71 (1.13, 2.59) | 0.01 | 1.67 (0.97, 2.9) | 0.07 | 1.23 (0.76, 1.99) | 0.40 | 2.06 (1.38, 3.08) | < 0.01 |
| Vaccine benefit |  |  |  |  |  |  |  |  |  |  |  |  |  |  |
| High | Reference |  |  |  |  |  |  |  |  |  |  |  |  |  |
| Low | 26.6 (8.77, 80.68) | < 0.01 | 9.18 (4.33, 19.46) | < 0.01 | 6.75 (3.46, 13.2) | < 0.01 | 19.14 (7.73, 47.41) | < 0.01 | 24.11 (9.85, 59.01) | < 0.01 | 19.17 (8.47, 43.37) | < 0.01 | 9.85 (5.69, 17.06) | < 0.01 |
| Trust in health authorities |  |  |  |  |  |  |  |  |  |  |  |  |  |  |
| High | Reference |  |  |  |  |  |  |  |  |  |  |  |  |  |
| Low | 25.2 (11.28, 56.31) | < 0.01 | 15.67 (8.34, 29.42) | < 0.01 | 27.85 (13.16, 58.94) | < 0.01 | 22.13 (10.92, 44.83) | < 0.01 | 22.36 (10.51, 47.55) | < 0.01 | 29.91 (14.96, 59.79) | < 0.01 | 12.61 (7.68, 20.71) | < 0.01 |
| Trust in the government’s preventive capacity | |  |  |  |  |  |  |  |  |  |  |  |  |  |
| High | Reference |  |  |  |  |  |  |  |  |  |  |  |  |  |
| Low | 1.06 (0.57, 1.96) | 0.86 | 1.32 (0.79, 2.19) | 0.29 | 1.12 (0.68, 1.83) | 0.66 | 1.47 (0.9, 2.42) | 0.12 | 4.14 (2.5, 6.87) | < 0.01 | 1.22 (0.71, 2.09) | 0.48 | 1.09 (0.68, 1.75) | 0.73 |
| Preference for infection–derived immunity | |  |  |  |  |  |  |  |  |  |  |  |  |  |
| High | Reference |  |  |  |  |  |  |  |  |  |  |  |  |  |
| Low | 3.65 (2.33, 5.74) | < 0.01 | 2.58 (1.74, 3.8) | < 0.01 | 1.72 (1.06, 2.77) | 0.03 | 2.38 (1.59, 3.55) | < 0.01 | 1.77 (1.08, 2.89) | 0.02 | 3.41 (2.1, 5.53) | < 0.01 | 2.64 (1.7, 4.09) | < 0.01 |

^#^: Full COVID–19 vaccination was defined according to national guidelines as receipt of at least three doses of an inactivated COVID–19 vaccine during pandemic period.

Table S10. Adjusted odds ratios for potential factors associated with pre–season influenza vaccine uptake across seven Chinese megacities

| Variables | Beijing | | Shanghai | | Guangzhou | | Shenzhen | | Tianjin | | Chongqing | | Chengdu | |
| --- | --- | --- | --- | --- | --- | --- | --- | --- | --- | --- | --- | --- | --- | --- |
|  | *aOR* (95% *CI*) | *P*–value | *aOR* (95% *CI*) | *P* value | *aOR* (95% *CI*) | *P*–value | *aOR* (95% *CI*) | *P*–value | *aOR* (95% *CI*) | *P*–value | *aOR* (95% *CI*) | *P*–value | *aOR* (95% *CI*) | *P*–value |
| Sex |  |  |  |  |  |  |  |  |  |  |  |  |  |  |
| Male | Reference | |  |  |  |  |  |  |  |  |  |  |  |  |
| Female | 0.78 (0.59, 1.02) | 0.07 | 1.23 (0.86, 1.77) | 0.26 | 1.06 (0.82, 1.37) | 0.67 | 1.13 (0.84, 1.52) | 0.41 | 1.23 (0.96, 1.58) | 0.11 | 0.98 (0.74, 1.29) | 0.86 | 1.3 (1.01, 1.66) | 0.04 |
| Age group | |  |  |  |  |  |  |  |  |  |  |  |  |  |
| Young adults (18–44 years) | Reference | |  |  |  |  |  |  |  |  |  |  |  |  |
| Adults (45–64 years) | 0.47 (0.33, 0.66) | < 0.01 | 1.04 (0.68, 1.6) | 0.86 | 1.06 (0.77, 1.44) | 0.73 | 1.14 (0.77, 1.7) | 0.50 | 1.33 (0.98, 1.8) | 0.07 | 1.09 (0.79, 1.5) | 0.61 | 1.29 (0.96, 1.71) | 0.09 |
| Older adults (≥ 65 years) | 1.74 (1.02, 2.98) | 0.04 | 1.18 (0.6, 2.34) | 0.63 | 1.34 (0.75, 2.42) | 0.32 | 3.7 (1.89, 7.22) | < 0.01 | 1.95 (1.23, 3.1) | < 0.01 | 1.44 (0.88, 2.35) | 0.15 | 2.03 (1.31, 3.15) | < 0.01 |
| Residence type | |  |  |  |  |  |  |  |  |  |  |  |  |  |
| City center | Reference | |  |  |  |  |  |  |  |  |  |  |  |  |
| Suburban | 1.02 (0.76, 1.36) | 0.91 | 1.28 (0.84, 1.93) | 0.25 | 0.81 (0.61, 1.06) | 0.13 | 0.75 (0.55, 1.02) | 0.07 | 1.64 (1.14, 2.36) | < 0.01 | 1.2 (0.82, 1.77) | 0.35 | 1.13 (0.88, 1.44) | 0.34 |
| Outer suburban and other | 1.12 (0.72, 1.73) | 0.62 | 0.81 (0.5, 1.31) | 0.39 | 0.7 (0.47, 1.05) | 0.09 | 0.61 (0.3, 1.23) | 0.17 | 0.79 (0.55, 1.14) | 0.21 | 0.86 (0.33, 2.23) | 0.75 | 1.11 (0.67, 1.85) | 0.69 |
| Occupation | |  |  |  |  |  |  |  |  |  |  |  |  |  |
| Professional | Reference | |  |  |  |  |  |  |  |  |  |  |  |  |
| Clerical and service worker | 0.6 (0.41, 0.87) | < 0.01 | 0.63 (0.3, 1.32) | 0.22 | 1.31 (0.93, 1.84) | 0.13 | 0.4 (0.28, 0.58) | < 0.01 | 0.56 (0.4, 0.79) | < 0.01 | 0.47 (0.33, 0.68) | < 0.01 | 1.08 (0.77, 1.51) | 0.67 |
| Production worker | 0.54 (0.28, 1.02) | 0.06 | 0.68 (0.26, 1.78) | 0.43 | 0.98 (0.59, 1.64) | 0.94 | 0.57 (0.31, 1.05) | 0.07 | 0.59 (0.38, 0.9) | 0.02 | 0.54 (0.34, 0.86) | < 0.01 | 1.06 (0.7, 1.6) | 0.79 |
| Student | 1.26 (0.5, 3.13) | 0.63 | 0.47 (0.15, 1.51) | 0.20 | 0.96 (0.47, 1.99) | 0.92 | 0.72 (0.31, 1.67) | 0.45 | 1.3 (0.66, 2.54) | 0.44 | 1.23 (0.54, 2.83) | 0.62 | 2.4 (1.01, 5.71) | 0.05 |
| Homemaker/Retired/Not employed nor schooling | 0.69 (0.4, 1.2) | 0.19 | 0.57 (0.25, 1.3) | 0.18 | 0.93 (0.56, 1.55) | 0.78 | 0.47 (0.28, 0.81) | < 0.01 | 0.3 (0.19, 0.47) | < 0.01 | 0.64 (0.41, 1.01) | 0.06 | 1 (0.65, 1.54) | 0.99 |
| Other | 0.5 (0.22, 1.13) | 0.10 | 0.81 (0.3, 2.2) | 0.68 | 1.41 (0.53, 3.75) | 0.49 | 0.4 (0.21, 0.78) | < 0.01 | 0.54 (0.27, 1.08) | 0.08 | 0.59 (0.31, 1.15) | 0.12 | 0.56 (0.31, 1.04) | 0.07 |
| Annual income | |  |  |  |  |  |  |  |  |  |  |  |  |  |
| < 10,000 CNY | Reference | |  |  |  |  |  |  |  |  |  |  |  |  |
| 10,000 – 49,999 CNY | 1.38 (0.89, 2.15) | 0.15 | 0.45 (0.23, 0.89) | 0.02 | 0.75 (0.5, 1.14) | 0.18 | 1.02 (0.64, 1.61) | 0.93 | 0.81 (0.57, 1.16) | 0.25 | 0.82 (0.54, 1.25) | 0.36 | 1.07 (0.77, 1.49) | 0.69 |
| 50,000 – 99,999 CNY | 1.37 (0.88, 2.13) | 0.16 | 0.58 (0.3, 1.11) | 0.10 | 0.77 (0.51, 1.16) | 0.22 | 0.84 (0.52, 1.36) | 0.48 | 1.01 (0.69, 1.49) | 0.95 | 0.96 (0.62, 1.47) | 0.84 | 1.14 (0.79, 1.65) | 0.47 |
| 100,000 – 199,999 CNY | 1.5 (0.94, 2.39) | 0.09 | 0.65 (0.34, 1.24) | 0.19 | 0.84 (0.55, 1.3) | 0.43 | 0.78 (0.47, 1.28) | 0.33 | 0.86 (0.55, 1.34) | 0.49 | 1.34 (0.81, 2.22) | 0.26 | 1.01 (0.61, 1.69) | 0.97 |
| 200,000 – 499,999 CNY | 1.4 (0.68, 2.87) | 0.36 | 1.28 (0.57, 2.91) | 0.55 | 0.56 (0.32, 0.99) | 0.05 | 0.85 (0.51, 1.43) | 0.54 | 1.13 (0.43, 2.94) | 0.81 | 1.47 (0.43, 5.05) | 0.54 | 1.33 (0.58, 3.05) | 0.50 |
| ≥ 500,000 CNY | 3.56 (1.34, 9.52) | 0.01 | 1.66 (0.6, 4.59) | 0.33 | 1.66 (0.58, 4.77) | 0.35 | 0.79 (0.32, 1.93) | 0.60 | 1.92 (0.77, 4.78) | 0.16 | 1.2 (0.36, 3.97) | 0.77 | 0.72 (0.27, 1.92) | 0.52 |
| Unclear/Unknown | 2.17 (1.08, 4.37) | 0.03 | 1.16 (0.46, 2.93) | 0.75 | 1.46 (0.73, 2.9) | 0.28 | 0.83 (0.43, 1.6) | 0.58 | 1.14 (0.68, 1.93) | 0.62 | 1.12 (0.57, 2.18) | 0.75 | 0.86 (0.51, 1.45) | 0.56 |
| Chronic Medical condition | | |  |  |  |  |  |  |  |  |  |  |  |  |
| Yes | Reference | |  |  |  |  |  |  |  |  |  |  |  |  |
| No | 1.00 (0.69, 1.47) | 0.98 | 0.80 (0.52, 1.24) | 0.32 | 1.19 (0.81, 1.75) | 0.38 | 1.24 (0.77, 2) | 0.37 | 0.84 (0.59, 1.2) | 0.34 | 0.67 (0.46, 0.98) | 0.04 | 0.94 (0.65, 1.35) | 0.74 |
| Self–rated health | |  |  |  |  |  |  |  |  |  |  |  |  |  |
| Poor | Reference | |  |  |  |  |  |  |  |  |  |  |  |  |
| Fair | 0.72 (0.35, 1.47) | 0.37 | 0.87 (0.36, 2.12) | 0.77 | 1.78 (0.93, 3.41) | 0.08 | 1.27 (0.61, 2.64) | 0.53 | 0.77 (0.39, 1.51) | 0.45 | 1.1 (0.52, 2.34) | 0.79 | 1.39 (0.68, 2.83) | 0.37 |
| Good | 1 (0.64, 1.57) | 1.00 | 0.96 (0.55, 1.68) | 0.88 | 0.8 (0.53, 1.2) | 0.28 | 0.97 (0.61, 1.54) | 0.90 | 1.36 (0.89, 2.06) | 0.15 | 0.83 (0.52, 1.32) | 0.43 | 0.81 (0.52, 1.25) | 0.34 |
| Living status | |  |  |  |  |  |  |  |  |  |  |  |  |  |
| Living alone | Reference | |  |  |  |  |  |  |  |  |  |  |  |  |
| Living with others | 1.26 (0.59, 2.69) | 0.55 | 1.33 (0.79, 2.21) | 0.28 | 1.51 (0.92, 2.49) | 0.11 | 1.03 (0.56, 1.89) | 0.92 | 0.9 (0.49, 1.63) | 0.72 | 0.77 (0.44, 1.35) | 0.36 | 1.46 (0.79, 2.71) | 0.23 |
| Fully vaccinated against COVID–19 | | | |  |  |  |  |  |  |  |  |  |  |  |
| Yes | Reference | |  |  |  |  |  |  |  |  |  |  |  |  |
| No | 0.24 (0.14, 0.44) | < 0.01 | 0.33 (0.16, 0.66) | < 0.01 | 0.44 (0.27, 0.73) | < 0.01 | 0.32 (0.17, 0.6) | < 0.01 | 0.22 (0.11, 0.44) | < 0.01 | 0.65 (0.36, 1.17) | 0.15 | 0.3 (0.18, 0.5) | < 0.01 |
| Perceived infection risk of influenza | | | |  |  |  |  |  |  |  |  |  |  |  |
| Low | Reference | |  |  |  |  |  |  |  |  |  |  |  |  |
| Medium | 1.44 (1.07, 1.95) | 0.02 | 1.01 (0.67, 1.5) | 0.97 | 0.94 (0.7, 1.25) | 0.65 | 0.88 (0.64, 1.2) | 0.41 | 0.8 (0.6, 1.05) | 0.11 | 1.02 (0.75, 1.37) | 0.91 | 0.99 (0.78, 1.27) | 0.95 |
| High | 1.25 (0.98, 1.6) | 0.07 | 1.18 (0.87, 1.61) | 0.29 | 1.24 (1, 1.55) | 0.05 | 1.07 (0.84, 1.36) | 0.58 | 0.97 (0.78, 1.2) | 0.75 | 1.28 (1.01, 1.63) | 0.04 | 0.98 (0.81, 1.2) | 0.86 |
| Knowledge of influenza vaccine | | | |  |  |  |  |  |  |  |  |  |  |  |
| High | Reference | |  |  |  |  |  |  |  |  |  |  |  |  |
| Low | 0.51 (0.33, 0.78) | < 0.01 | 0.48 (0.29, 0.77) | < 0.01 | 0.66 (0.48, 0.9) | < 0.01 | 0.54 (0.39, 0.76) | < 0.01 | 0.72 (0.51, 1.02) | 0.07 | 0.54 (0.37, 0.78) | < 0.01 | 0.74 (0.53, 1.02) | 0.07 |
| Vaccine confidence | |  |  |  |  |  |  |  |  |  |  |  |  |  |
| High | Reference | |  |  |  |  |  |  |  |  |  |  |  |  |
| Low | 1.28 (0.87, 1.88) | 0.22 | 0.87 (0.53, 1.45) | 0.60 | 0.79 (0.56, 1.1) | 0.16 | 0.7 (0.49, 0.99) | 0.04 | 1.1 (0.76, 1.6) | 0.60 | 0.81 (0.56, 1.16) | 0.25 | 0.84 (0.63, 1.13) | 0.26 |
| Vaccine benefit | |  |  |  |  |  |  |  |  |  |  |  |  |  |
| High | Reference | |  |  |  |  |  |  |  |  |  |  |  |  |
| Low | 0.95 (0.55, 1.62) | 0.84 | 1.17 (0.67, 2.04) | 0.58 | 1.12 (0.69, 1.83) | 0.65 | 1.25 (0.76, 2.07) | 0.38 | 0.89 (0.54, 1.47) | 0.65 | 1.03 (0.64, 1.68) | 0.90 | 1.04 (0.7, 1.54) | 0.86 |
| Trust in health authorities | |  |  |  |  |  |  |  |  |  |  |  |  |  |
| High | Reference | |  |  |  |  |  |  |  |  |  |  |  |  |
| Low | 0.45 (0.27, 0.75) | p < 0.01 | 0.44 (0.26, 0.75) | < 0.01 | 0.57 (0.36, 0.9) | 0.02 | 0.48 (0.3, 0.77) | < 0.01 | 0.83 (0.51, 1.35) | 0.45 | 0.91 (0.58, 1.45) | 0.70 | 0.51 (0.34, 0.75) | < 0.01 |
| Trust in the government’s preventive capacity | | |  |  |  |  |  |  |  |  |  |  |  |  |
| High | Reference | |  |  |  |  |  |  |  |  |  |  |  |  |
| Low | 0.9 (0.56, 1.44) | 0.66 | 1.25 (0.76, 2.05) | 0.39 | 0.95 (0.64, 1.42) | 0.80 | 1.3 (0.86, 1.95) | 0.21 | 0.94 (0.64, 1.39) | 0.77 | 0.84 (0.56, 1.26) | 0.41 | 1.55 (1.12, 2.15) | < 0.01 |
| Preference for infection–derived immunity | | |  |  |  |  |  |  |  |  |  |  |  |  |
| High | Reference | |  |  |  |  |  |  |  |  |  |  |  |  |
| Low | 0.83 (0.59, 1.18) | 0.31 | 0.91 (0.6, 1.38) | 0.65 | 0.83 (0.6, 1.16) | 0.28 | 0.77 (0.56, 1.08) | 0.13 | 0.94 (0.68, 1.29) | 0.69 | 0.56 (0.4, 0.78) | < 0.01 | 0.64 (0.48, 0.86) | < 0.01 |

^#^: Full COVID–19 vaccination was defined according to national guidelines as receipt of at least three doses of an inactivated COVID–19 vaccine during pandemic period.

Table S11. Sensitivity analysis of factors associated with influenza vaccine hesitancy using alternative hesitancy classification (neutral responses classified as non–hesitant)

| Variable | *aOR* (95% *CI*) | *P*–value |
| --- | --- | --- |
| Sex |  |  |
| Male | Reference |  |
| Female | 0.77 (0.64, 0.92) | < 0.01 |
| Age group |  |  |
| Young adults (18–44 years) | Reference |  |
| Adults (45–64 years) | 0.95 (0.77, 1.18) | 0.65 |
| Older adults (≥ 65 years) | 0.75 (0.54, 1.05) | 0.1 |
| Residence type |  |  |
| City center | Reference |  |
| Outer suburban | 0.84 (0.69, 1.02) | 0.08 |
| Suburban and other | 1.3 (1.03, 1.64) | 0.03 |
| Occupation |  |  |
| Professional | Reference |  |
| Clerical and service worker | 2.21 (1.64, 2.97) | < 0.01 |
| Production worker | 2.37 (1.66, 3.39) | < 0.01 |
| Student | 1.76 (1.00, 3.09) | 0.05 |
| Homemaker/Retired/Not employed nor schooling | 1.97 (1.36, 2.85) | < 0.01 |
| Other | 2.78 (1.82, 4.25) | < 0.01 |
| Annual income |  |  |
| < 10,000 CNY | Reference |  |
| 10,000 – 49,999 CNY | 1.25 (0.96, 1.62) | 0.1 |
| 50,000 – 99,999 CNY | 1.04 (0.78, 1.37) | 0.8 |
| 100,000 – 199,999 CNY | 1.09 (0.80, 1.50) | 0.58 |
| 200,000 – 499,999 CNY | 1.03 (0.66, 1.60) | 0.91 |
| ≥ 500,000 CNY | 0.67 (0.30, 1.49) | 0.32 |
| Unclear/Unknown | 1.00 (0.67, 1.48) | 0.99 |
| Chronic Medical condition |  |  |
| Yes | Reference |  |
| No | 0.90 (0.70, 1.15) | 0.39 |
| Self–rated health |  |  |
| Poor | Reference |  |
| Fair | 0.60 (0.43, 0.84) | < 0.01 |
| Good | 1.25 (0.99, 1.56) | 0.06 |
| Living status |  |  |
| Living alone | Reference |  |
| Living with others | 0.80 (0.58, 1.10) | 0.17 |
| Fully COVID–19 vaccination* |  |  |
| Yes | Reference |  |
| No | 1.42 (1.09, 1.85) | < 0.01 |
| Perceived infection risk of influenza |  |  |
| Low | Reference |  |
| Medium | 0.60 (0.50, 0.72) | < 0.01 |
| High | 1.24 (1.07, 1.44) | < 0.01 |
| Knowledge of influenza vaccine |  |  |
| High | Reference |  |
| Low | 0.26 (0.21, 0.33) | < 0.01 |
| Vaccine confidence |  |  |
| High | Reference |  |
| Low | 1.20 (0.92, 1.58) | 0.18 |
| Perceived vaccine benefit |  |  |
| High | Reference |  |
| Low | 2.08 (1.45, 2.98) | < 0.01 |
| Trust in health authorities |  |  |
| High | Reference |  |
| Low | 5.28 (3.26, 7.71) | < 0.01 |
| Trust in government’s preventive capacity |  |  |
| High | Reference |  |
| Low | 0.46 (0.34, 0.64) | < 0.01 |
| Preference for infection–derived immunity |  |  |
| High | Reference |  |
| Low | 0.54 (0.41, 0.71) | < 0.01 |

^*^: Full COVID–19 vaccination was defined according to national guidelines as receipt of at least three doses of an inactivated COVID–19 vaccine during pandemic period.

Figure S1. Sampling strategy for the cross–sectional survey on influenza vaccine hesitancy in seven Chinese megacities
